# Supplementary material for: miR-103 promotes endothelial maladaptation by targeting lncWDR59
Source: Nat Commun. 2018 Jul 6;9:2645. doi: 10.1038/s41467-018-05065-z (PMC6035258; doi:10.1038/s41467-018-05065-z)
Supplement: Supplementary file 1 — Supplementary Information [file 41467_2018_5065_MOESM1_ESM.pdf]

Supplementary Information

miR-103 promotes endothelial maladaptation by targeting lncWDR59. Natarelli L. et al.

Supplementary methods

Animal models. *Apoe*$^{-/-}$ mice (The Jackson Laboratory, Bar Harbor, ME, USA) were used for experiments. Cdh5-CreER^{T2}Dicer1$^{flox/flox}$/Apoe$^{-/-}$ (EC-Dicer^{flox}) and Cdh5-CreER^{T2}Dicer1$^{WT/WT}$/Apoe$^{-/-}$ (EC-Dicer^{WT}) littermates were used for experiments. Deletion of the conditional *Dicer* allele was induced by intraperitoneal injection of tamoxifen[1]. One week after the last TMX injection, 6- to 8-week-old mice were fed a high-fat diet (HFD), comprising 21% crude fat, 0.15% cholesterol, and 19.5% protein for 12 weeks. *Apoe*$^{-/-}$ mice, 6- to 8-week-old, were instead fed a chow-diet (normal diet, ND), comprising 3.3% crude fat and 19% protein, or a HFD, for 4 or 12 weeks. All animal experiments were reviewed and approved by the local authorities (State Agency for Nature, Environment and Consumer Protection of North Rhein-Westphalia and District Government of Upper Bavaria) in accordance with German animal protection laws. Tissues were paxgene or 4% paraformaldehyde (PFA) fixed and paraffin embedded for further experiments. All tissues were stored at -20°C to preserve the RNA integrity.

Global Gene Expression Analysis of lncRNAs. Arteries from EC-Dicer^{flox} and EC-Dicer^{WT} mice (n=2 per group) were excised after perfusion with RNAlater (Life Technologies, Darmstadt, Germany). Global gene expression analysis was performed using 8 × 60K SurePrint G3 Mouse Gene Expression (Agilent, Design ID 028005) in combination with a one-color based hybridization protocol (IMGM Laboratories GmbH, Munich, Germany)[1]. Agilent AMADID 028005 probes for lncRNA detection were designed in collaboration with the Broad institute and according to followed databases: RefSeq Build 37, Ensembl Release 55, Unigene Build 176, GenBank (April 2009), and RIKEN 3. Fluorescent signals on the microarrays were detected using the Agilent DNA Microarray Scanner (Agilent Technologies Germany GmbH, Böblingen, Germany).

First, lncRNAs probes not detectable in any of the four samples were flagged.

Since the majority of the lncRNAs were detected by more than one probe, lncRNA probe redundancy was eliminated by performing a “probe-to-gene analysis” to generate a final non-redundant lncRNA gene list. Briefly, probes for each lncRNA were ranked according to 3 parameters: detection of the probe, *P*-value and fold change. Redundant lncRNAs with the same predicted, strand-specific, genomic coordinates (e.g., lincRNA:chr8:113968152-113973727 reverse strand) were ranked as 1 putative lncRNA and analyzed as follow: among those putative lncRNAs detected by n≥3 probes, a number of positively detected probes ≥2 was considered acceptable to confirm the significance of lncRNA detection. After that, we selected the one with a P-value and fold change closed to the average calculated as follow:

$$PA = (x\text{Probe}_1 + x\text{probe}_2 +..x\text{probe}_n)\ n^{-1}$$
$$FCA = (y\text{Probe}_1 + y\text{probe}_2 +..y\text{probe}_n)\ n^{-1}$$

Were PA is the P-value average, FCA is the fold change average, x is the P-value of probe$_n$, y the fold change of probe$_n$ and n the number of probes identifying the putative lncRNA.

For all putative lncRNAs detected by n=2 probes, positive detection of one probe was considered acceptable to define the lncRNA as detectable and used as representative of the lncRNA expression. If both probes were detectable, the one with the lowest P-value and highest fold change was considered representative of the putative lncRNA expression. The “probe-to-gene analysis” generated a final list of 2926 non-redundant lncRNAs. Differential

expression of lncRNAs was determined by t-test using a *P*-value cutoff of 0.05 and a EC-Dicer^{flox}/EC-Dicer^{WT} ratio of 1.5.
Genomic localization of upregulated LncRNA was determined by Blast alignment of probes against the murine genome (following database conversion from mm8 to mm10) and consent the identification of the intergenic lncRNAs.

Immunostaining of murine tissues. Mice were anesthetized with ketamine (80 mg kg^{-1}) and medetomidine (0.3 mg kg^{-1}). After perfusion with PBS from left ventricle, mice were *in situ* perfused with paxgene (PAXgene, Qiagen GmbH, Hilden, Germany) or 4% PFA fixatives. The heart, aortic arch and thoraco-abdominal aorta were subsequently collected and paraffin embedded. Serial sections (5 µm thick) were collected. Immunofluorescence staining was performed by blocking sections for 1 h with a blocking solution of PBS containing 1% BSA and 1.25% normal horse serum. Primary antibodies were diluted in a solution of PBS containing 1% of blocking solution (Ab sol.) and incubated overnight to detect Ki67 (0.46 µg ml^{-1}, ab15580; Abcam), activated-Notch1 (8µg ml^{-1}, Notch intracellular domain, NICD, ab8925; Abcam), β-catenin (1 µg ml^{-1}, ab16051; Abcam), phospho-gamma H2AX for premature-senescence associated DNA damage (γH2AX, 1:250 dilution, A300-081A-M, Biomol), anti-GRO alpha (5 µg ml^{-1}, ab86436; Abcam) von Willebrand Factor (7,4 µg ml^{-1}, vWF, ab6994; Abcam) and PECAM-1 (M-20) (2,7 µg ml^{-1}, CD31, sc-1506; Santa Cruz). Nonspecific primary antibodies were used as negative controls (Santa Cruz Biotechnology). Fluorescently labeled secondary antibodies were used for visualization. Cell nuclei were counterstained with 4',6-diamidino-2-phenylindole (DAPI; Vectashield, Vector Laboratories, Peterborough, UK). Digital images were acquired using a Leica DM6000B fluorescent microscope (Leica Microsystems GmbH, Wetzlar, Germany) equipped with a digital camera (Leica DFC365 FX, Leica Microsystem). For quantification, 3-4 sections *per* mouse were used and quantification was performed with ImageJ. Data were expressed as number of vWF or PECAM-1 positive cells showing a Ki67, NICD, β-catenin or γH2AX nuclear localization normalized on total number of vWF or PECAM-1 positive cells and expressed in percentage.
To study micronuclei (MN) formation and DNA-damage accumulation at predilection and non-predilection sites, aortic arches and thoracic aortas were *en face* prepared and stained for CD31 and γH2AX. Cell nuclei were counterstained with DAPI. For quantification, *en face* from 4-6 mice *per* group were entirely quantified.

RNA fluorescence *in situ* hybridization (FISH) on human and murine tissues
RNA FISH assay was performed using the Affymetrix protocol (ViewRNA Cell Plus Assay, Affymetrix) with some modifications. Briefly, 5µm paxgene fixed/paraffin embedded sections freshly cutted and stored at -20°C to preserve RNA integrity, were de-paraffinized and boiled for antigen retrival in a citrate buffer solution and incubated with RNase-free DNase (Roche Diagnostics, 10776785001) at 37°C for 15 h for DNase digestion. Tissues were washed in RNase-free water and ethanol, dried and incubated with custom probe oligonucleotides specific for miR-103 and lncWDR59 designed and synthesized by Affymetrix as Type 1 and 6, respectively, following the manufacturer instructions. At the end of the hybridization, sections were blocked for 1 h with 1%-BSA-blocking solution (5,4ml PBS + 600µl 10% BSA + 75µl normal horse serum), then incubated with anti-vWF antibody overnight in a humid chamber. Fluorescently labeled (FITC-labeled) secondary antibody was used for vWF visualization. Cell nuclei were counterstained with DAPI. Digital images were acquired as described before.

Laser-Capture Microdissection (LCM). The roots were harvested from *Apoe*$^{-/-}$ mice after a 4-week HFD feeding period, manually paxgene-fixed and embedded in paraffin. Serial sections (5 µm thick) were collected on UV-sterilized and RNase-free polyester-membrane 0.9 µm FrameSlides (Leica), deparaffinized under RNase-free conditions and completely dried at 40°C. ECs and plaques were collected using a laser microdissection system (LMD7000, Leica) in RNase-free tubes. RNA was isolated with the PAXgene RNA MinElute kit (Qiagen) and followed by pre-amplification and reverse transcription with Ovation PicoSL WTA System V2 (NuGEN) following manufacturer's instructions.

RNA isolation and next-generation sequencing (RNA-seq). Primary murine aortic endothelial cells (MAoECs; passage 3; PELOBiotech GmbH, Planneg, Germany) were cultured using endothelial cell complete growth medium (Promocell) containing gentamicin (0.05 mg/mL; ThermoFisher). Total RNA was isolated using the NucleoSpin microRNA Kit (Macherey-Nagel GmbH & Co. KG). RNA purity and integrity were assessed using the Fragment Analyzer™ Automated CE System (Advanced Analytical, Heidelberg, Germany). A RQN of 8.8 and a 28S/18S ratio of 2.2 were considered acceptable for next generation sequencing assay. Five µg of DNase-treated RNA were used to prepare Massive Analysis of cDNA ends (MACE) libraries needed to perform a DNA-Methylation-Sequencing (Meth-Seq) PCR bias free quantification with TrueQuant Technology, followed by a high-throughput sequencing on the Illumina Genome Analyzer II system (GenXPro GmbH, Frankfurt, Germany). The procedure consist in the extraction of poly-adenylated RNA from 5 µg RNA and reverse transcribed with biotinylated poly(T) primers. cDNA is fragmented to an average size of 250 bp. Biotinylated ends are captured by streptavidin beads and ligated to modified adapters (TrueQuant DNA adapter, GenXPro). The libraries are amplified by PCR, purified by SPRI beads and sequenced (2 x 100 bp Illumina HiSeq2000 TrueSeq, 2 x 20 Mio. Reads poly-A selected paired-end reads). Paired end sequencing of both DNA strands from each end is required for fragment strand specificity.

Bioinformatics analysis of RNA-seq data. Bioinformatics analysis was performed following a trimming and quality assessment of sequences (Tophat/Cufflinks) through a TrueQuant elimination of PCR artifacts, followed by a strand specific mapping of sequencing reads to online available databases, analysis of differential expression (p-value), and identification of annotated lncRNAs and new lncRNAs using strand specific fragments. A maximum of 5% overlapping between lncRNA fragments and protein-coding genes was used as cut-off to determine new lncRNA genes. RNA-seq data were also compared with those from SRA dataset for transcriptome of the main olfactory epithelium (MOE) of *Castaneus mice* at 12 weeks (SRA: ERR657457). Strand specific datasets were also used to find strand-specific data at all. The gene-wise fold changes for the non-ribosomal genes were calculated, based on strand-specific mappings where both fragments were mapped within the gene. Among genes with a high number of read fragments there were endothelial-specific marker genes, such as *Cdh5*, *Nos3*, *Pecam1*, *Ctnnb1* and *Notch4*, together with *Malat1*, *Neat1*, *Pvt1* and *Fendrr*, known endothelial lncRNAs (**Supplementary Figure 1A-B**).

Analysis of the DNA methylation state for murine and human *lncWDR59*, and *Leonardo*. Histone modification signatures of genes actively transcribed by RNA polymerase II, such as trimethylation state on histone 3 of lysine 36 (H3K36m3), lysine 4 (H3K4m3), and lysine 27 (H3K27me3) and the acetylation of lysine 27 (H3K27ac) were used to identify the

transcript and promoter regions of lncRNAs, respectively. In particular, H3K4m3, H3K27ac and H3K27me3 signatures were used to identify active promoter and enhancer regions (intergenic K4-K36 domains), while H3K36m3 markers identify transcript regions. Database described from Guttman M. et al.[2] and Chip-seq from Mikkelsen, Xu et al. (2010) were used for analysis and visualized using an Integrative Genomics Viewer interface (**Supplementary Fig. 1D,E**).

Hot Start-Rapid Amplification of cDNA Ends PCR (Hot Start-RACE PCR). To determine the sequence of lncWDR59, 3´/5´ RACE PCR was performed using the 5´/3´RACE Amplification kit, 2nd Generation (Roche) with some modifications due to the complexity of lncRNA secondary structure. First, after assessing RNA purity and integrity as mentioned above, we designed primers flanking the probe sequence used for Global Gene Expression Analysis (**Supplementary Table 1** and **Supplementary Figure 1E**). The sequence identity of lncWDR59 amplicon was confirmed by Sanger sequencing (Eurofins Genomics, Munich, Germany). To increase the efficiency of the 3´ end cDNA synthesis, we performed a Hot-start first strand synthesis set up in order to open complex secondary structures but optimized to avoid the degradation of the RNA. One µg of total RNA was incubated with a dNTP mixture, oligo dT-anchor primers (both from Roche kit), and 5X PrimeScript Buffer (Clonetech/Takara, Code No. 2680Q) in a thermocycler. A control RNA (from Roche kit) was used as positive control in all steps. As soon as the temperature reached 50°C, RNase inhibitor (from Roche kit) and PrimeScript Reverse Transcriptase (Clonetech/Takara) were added to the samples and the reaction was performed as follow: 10 min at 70°C, 1 h at 55°C, and a final step of 5 minutes at 85°C to inactivate the Reverse Transcriptase. 3´cDNA end synthesis was performed using specific SP5 forward primers designed according to the tri-methylation state of K36 domains as follow: closed to the probe sequence, after the probe sequence, and closed to the predicted 3´end (all 12.5 µM) (**Supplementary Table 1** and **Supplementary Figure 1E**). The reaction was prepared using 1µl of cDNA, a PCR anchor primer, a dNTP mixture (all from Roche kit), and the Phusion® High-Fidelity DNA Polymerase (0.02 U μl^{-1}) together with the 5X Phusion GC Buffer (both from New England BioLabs). Single primer reactions were also run as control to exclude possible nonspecific PCR products. As positive control, a neo3/for primer (from Roche kit) was used. PCR was performed as follow: 2 min at 98°C; 35 cycles 15 sec at 98°C, 1 min at 55°C, 40 sec at 72°C; 1 cycle of 7 min at 72°C. PCR amplification products were loaded on a 1% agarose gel. The 3 visible bands were catted and isolated from the agarose gel using the Gel extraction kit (Qiagen). The cDNA from the ~1300 and ~900 bp band was re-amplified, run on 1% agarose gel and sent for sequencing (Eurofins Genomics, Munich, Germany). 5´RACE PCR was performed using the 5´/3´RACE Amplification kit, 2nd Generation (Roche), according to manufactures' instructions, using a specific SP1 primer, the Phusion® High-Fidelity DNA Polymerase (0.02 U/µl), and a SP2 primer for a secondary nested amplification step (**Supplementary Table 1** and **Supplementary Figure 1E**). As control a neo1/rev primer (from Roche kit) was used. The weak band at ~200 and ~100 bp bands generated with SP1 and SP2 primers, respectively, were isolated, re-amplified and sent for sequencing (Eurofins).

To further confirm the sequence of the lncWDR59 identified by 3´RACE, two primers within the newly discovered sequence were designed (middle Fwd and Rev primers in **Supplementary Table 1** and **Supplementary Figure 1E**). cDNA from Hot-start first strand cDNA synthesis was used to perform a PCR as follow: 2 min at 98°C; 35 cycles 15 sec at 98°C, 30 sec at 59°C, 40 sec at 72°C; 1 cycle of 4 min at 72°C; 4°C hold. PCR amplification product was loaded on a 2% agarose gel, isolated and sent for sequencing (Eurofins).

Sequencing confirmed the 1.152 kb PCR product length (from a 1.5 kb total transcript sequence).

Prediction of miRNA-binding on lncRNA transcripts. The miRNA target sites were predicted in those lncRNAs that were upregulated (fold change cutoff > 1.5) in EC-Dicer^{flox} mice fed 12 weeks of HFD, searching for binding sites for the 20 miRNAs down-regulated in the same mice[1]. Prediction was performed using the RNAhybrid target prediction algorithm. Binding sites were predicted for all lncRNA transcripts with a defined sequence. We searched for a minimum of 5 hits per target with a -20 energy threshold cutoff. MiRNA target sites with Watson-Crick pairing were selected and classified as canonical and non-canonical as previously described[3]. We admitted only 1 G-U mismatch in seed-target bindings that were then classified as non-canonical. Finally, the miRNA-lncRNA interacting network was built with Cytoscape. Among the lncRNA putative targets of let-7b and miR-103, the expression of the lncRNAs containing BSs with a minimum free energy of at least -25 was analyzed in EC-Dicer^{flox} and EC-Dicer^{WT} mice by qPCR.

Prediction of lncRNA secondary structure. Secondary structure of Leonardo and lncWDR59 was predicted using RNAfold prediction algorithm. Functional RNA sequences on lncWDR59 were predicted using RegRNA 2.0 algorithm.

MicroRNA Target Identification and Quantification System (MirTrap). MAoECs were co-transfected with miR-103-mimics, let-7b mimics, or scrambled controls, and pMirTrap Vector using the XfectTM MicroRNA Transfection Reagent in combination with Xfect Polymer for 24 h (all from Clontech). The pMirTrap Vector expressed a DYKDDDDK-tagged GW182 protein, member of the active RISC complex, which enabled locking of the miRNA/mRNA complex into the RISC[4]. After 24 h, ECs were harvested and washed in ice-cold 1x phosphate-buffered saline (GE Healthcare Life Sciences), and then incubated in Lysis Buffer provided by the MirTrap System and supplemented with protease inhibitors (Complete Protease Inhibitor Cocktail Tablets; Roche). The cell lysates were centrifuged and part of the input RNA was extracted from the supernatant using the NucleoSpin RNA XS Kit (Macherey-Nagel GmbH & Co. KG). Anti-DYKDDDDK-conjugated magnetic beads were washed twice with 1X Lysis/Wash Buffer containing 1mM DTT, 0.1 unit μl^{-1} RNase inhibitor and protease inhibitors (Complete Protease Inhibitor Cocktail Tablets; Roche), and blocked for 3 h at 4°C with 1.25 mg ml^{-1} tRNA solution and 1.25 mg ml^{-1} BSA. To immunoprecipitate (IP) the DYKDDDDK-tagged RISC complex, blocked Anti-DYKDDDDK beads were incubated with the cell extract for 2 h at 4°C. Immobilization of the precipitates and subsequent RNA isolation was performed using the NucleoSpin RNA XS Kit (Macherey-Nagel GmbH & Co. KG). Reverse-transcription of input and IP samples were performed using a high-capacity cDNA reverse transcription kit (Life Technologies), followed by the amplification with gene-specific primers (**Supplementary Table 1**) and SYBR Green PCR Master Mix (Thermo Scientific). The fold enrichment was calculated according to manufacturer's instructions. Efficiency of transfection was determined by performing a control transfection using miR-132-mimic, the pMirTrap Vector, and the pMirTrap Control Vector, which expresses an AcGFP1 fluorescein protein containing a miR-132 target sequence. The efficient enrichment of AcGFP1 fluorescein protein in the RISC was confirmed and compared to that of a non-miR-132 target gene, such as Lef1 mRNA transcript.

Human aortic ECs (HAoECs; passage 2-3; Promocell, Heidelberg, Germany) were cultured using endothelial cell complete growth medium (Promocell) containing gentamicin

(0.05 mg mL^{-1}; ThermoFisher) and co-transfected with miR-103-mimics or scrambled controls, together with pMirTrap Vector using the XfectTM MicroRNA Transfection Reagent in combination with Xfect Polymer for 24 h (all from Clontech)[1] to assess hsa-lncWD59 enrichment as described before.

Cell Culture and transfection. MAoECs and HAoECs were cultured in 0.2% gelatin-coated wells (Corning) or chamber slides (Thermo Fisher Scientific) as described before. To growth the cells under different shear stresses, MAoECs were cultured in collagen coated-perfusion chambers (µ-Slides $VI^{0.4}$, ibidi GmbH, Martinsried, Germany) and exposed to high shear stress (10 dyne cm^{-2}) or low shear stress (5 dyne cm^{-2}) for 48 h generated by the perfusion with EC complete medium (ibidi Pump System, ibidi GmbH).

Lipofectamine2000 (Life Technologies) was used to transfect MAoECs for 24 h with locked nucleic acid (LNA)-miR-103 or LNA-let-7b inhibitors (50 nM, miRCURY LNA™ microRNA Inhibitors; Exiqon, Vedbaek, Denmark), miR-103 or let-7b mimics (50 nM, *mir*Vana™ mimics; Life Technologies), antisense oligonucleotides to block the interaction between miR-103 and lncWDR59 (target site blockers, TSB; 50 nM, miRCURY LNA™ microRNA Target Site Blockers; Exiqon), murine lncWDR59 or Sox17 GapmeRs (50 nM, LNA™ GapmeRs; Exiqon), and scrambled controls. Lipofectamine2000 was also used to transfect HAoECs with human lncWDR59 GapmeRs (50 nM, LNA™ GapmeRs; Exiqon) or scrambled controls for 48 h. RNA interference technology (siRNA) was used to generate specific knockdown of β-catenin expression (40 nM, Qiagen). Notch1 activation was inhibited by treatment of MAoECs with the γ-secretase inhibitor DAPT (20 µM, Selleck Chemicals).

To simulate *in vitro* a condition closed to *in vivo* atherosclerosis, MAoECs were stimulated with low (25µg/ml) or high (100µg ml^{-1}) doses of oxLDL, prepared as previously described[5], for different time points.

Murine aortic smooth muscle cells (mAoSMCs P2/3, PELOBiotech) were cultured in supplemented complete smooth muscle cell medium (PELOBiotech, PB-M2268) and seeded in gelatin-coated wells or chamber slides and treated with oxLDL or transfected with TSBs as described for MAoECs. Bone marrow-derived macrophages were from the femurs of $Apoe^{-/-}$ mice were harvested and cultured in DMEM/F12 supplemented with 10% FBS and 10% L929-conditioned medium. The macrophages were stimulated with oxLDL (100 µg ml^{-1}) or PBS for 72 h.

RNA isolation and quantitative Real-time PCR (qPCR). Total RNA was isolated from MAoECs and HAoECs using the NucleoSpin microRNA Kit as described above, while nuclear and cytoplasmic RNA was isolated from MAoECs using the Cytoplasmic and Nuclear RNA Purification Kit (Norgen Biotek Corp). RNA was reverse-transcribed with a high-capacity cDNA reverse transcription kit (Life Technologies) or Taqman MicroRNA Reverse transcription kit (Thermo Fisher Scientific). The mRNA qPCR was performed either with TaqMan gene expression assays and TaqMan Universal PCR Master Mix (both from Life Technologies) or with gene-specific primers (Sigma-Aldrich) and a SYBR Green Master Mix (Thermo Scientific) (**Supplementary Table 1**). The miRNA qPCR was performed with TaqMan MicroRNA Assays (Thermo Fisher Scientific). All qPCR experiments were run on a 7900HT real-time PCR system (Life Technologies). Data were normalized to single or multiple reference genes (sno-135 and RNU44 for murine and human miRNAs; GAPDH and B2M for mRNAs), scaled to the sample with the lowest expression using QbasePLUS software (Biogazelle NV, Zwijnaarde, Belgium), and logarithmically transformed (log_{10}).

Flow cytometry analysis of endothelial cell cycle. To analyze the phases of cell cycle MAoECs were plated in 6-well plates and transfected for 24 h with TSBs or scrambled controls as described above. Cells were collected and fixed for 30 minutes on ice with 70% ethanol. After that cell were washed 3 times in a PBS solution containing 2% FCS and then resuspended and incubated for 1 h at 37°C in a PBS solution containing 20μg ml^{-1} propidium iodide (PI, Sigma), 10μg ml^{-1} RNase and 0.1% Triton X-100 (Sigma). Cells were analyzed by flow cytometry (FACSCantoII, BD Biosciences) after appropriate calibration settings and data analyzed using Flowing software 2.

Analysis of EC proliferation upon different shear stress conditions. MAoECs were cultured on collagen coated-perfusion chambers (μ-Slides $VI^{0.4}$, ibidi GmbH, Martinsried, Germany) and exposed to high shear stress (10 dyne cm^{-2}) or low shear stress (5 dyne cm^{-2}) for 48 h generated by the perfusion with EC complete medium (ibidi Pump System, ibidi GmbH) containing 5-ethynyl-2'-deoxyuridine (EdU, 10 μM final conc., Click-iT® EdU Alexa Fluor® 488 Imaging Kit, life technologies) and TSBs (TSBs *in vivo* ready), or control LNAs (both 50 nM final conc., Exiqon). At the end of the experiments, ECs were stained according to manual instructions. A minimum number of 50 random images all along the chamber slide was collected and quantified as indicated before. Data were represented as number of EdU^+ cells on total number of cells, in percentage (n = 3-4 per group).

Immunostaining on murine and human aortic ECs. MAoECs and HAoECs were cultured on chamber slides and treated as described above. For immunofluorescence staining of β-catenin, cells were fixed and permeabilized using -20°C cold 100% methanol. After 10 minutes of incubation at -20°C, cells were washed using cold PBS++ (PBS containing 1mM $CaCl_2$ and 1mM $MgCl_2$) and incubated overnight in humid chambers with β-catenin primary antibody (2 μg ml^{-1}). For immunofluorescence staining of Notch1 (780 μg ml^{-1}), Ki67 (0,7 μg ml^{-1}) and γH2AX (2 μg ml^{-1}) cells were fixed in 2% PFA for 30 min and then permeabilized for 30 minutes using a PBS++ solution containing 1% BSA and 0.1% Triton X-100. Cells were then washed using cold PBS++ and incubated overnight with the specific primary antibodies. Nonspecific primary antibodies were used as negative controls (Santa Cruz Biotechnology). Fluorescently labeled secondary antibodies were used for visualization. Cell nuclei were counterstained with DAPI. Digital images were acquired using a Leica DM6000B fluorescent microscope. For quantification, 10-20 images per each replicate (n=4-8 per group) were used and quantification was performed with ImageJ. Data were expressed as number of Ki67, NICD or β-catenin nuclear positive cells normalized on total number of cells and expressed in percentage.

Micronuclei (MN) analysis. Cells treated as described before were stained with DAPI. The percentage of micronucleated cells was determined by microscopy. MN were defined as DNA aggregates separated from the primary nucleus in cells with a normal interphase nuclear morphology. Cells with an apoptotic appearance were excluded. Data were expressed as number of micronucleated cells normalized on total number of cells, in percentage. For micronucleic γH2AX analysis, the number of cells with MN and/or principal nuclei (PN) positive (MN^+PN^- + MN^+PN^+) was normalized on total number of micronucleated cells (previously normalized on total number of cells), and expressed in percentage. To detect the fluorescent signal into cells and micronuclei and to collect representative pictures, **z+t** stacks of three-dimensional images were recorded using a Leica TCS SP8 STED confocal microscope using a 63x, 1.30 numerical aperture HC PL APO CS2 glycerol objective (Leica Microsystem, Germany) and 3D reconstructed using the LAS-X 3D algorithm.

Prediction of lncWDR59-protein interaction. The prediction of lncWDR59 interaction with activated-Notch1 (NICD), deltex4, Numb, alphaprotein-1 alpha (aph-1α), presenilin (psen)-1 and -2, and nicastrin proteins was performed using the catRAPID[6] prediction algorithm (**Supplementary Figure 1F**). For a more specific prediction a Global Score analysis for long transcripts was performed. In particular, Numb and lncWDR59 sequences were uniformly divided into sub-regions and fragments processed for RNA interaction propensity and protein-RNA interaction propensity to identify the top Numb-lncWDR59 interaction sites (**Supplementary Figure 1F**).

NICD- and Numb-RNA immunoprecipitation (IP). To analyze the interaction of lncWDR59 with Notch1 (NICD) and Numb, and of NICD with Numb, MAoECs were transfected with miR-103 or control inhibitors for 24 h. Cells were collected and lysate in lysis buffer (10 mM HEPES pH7.0, 100 mM KCl, 5 mM MgCl2, NP40, 5 mM DTT, 250 U mL^{-1} RNase inhibitor, 400 µM Vanadyl Ribonucleoside Complexes, protease inhibitor cocktail). Magnetic beads (1.5 mg Magna ChIP™ Protein A+G Magnetic Beads, Millipore) were washed twice with wash buffer (50 mM Tris, 150 mM NaCl, 10mM $MgCl_2$, NP-40, 40 U mL^{-1} RNase inhibitor, 5 mM DTT) and incubated with rotation with 8µg of anti-Numb antibody (ABIN374158, Antibodies online), anti-activated Notch1 antibody (ab8925, abcam) or 8µg of IgG for 30 minutes at room temperature. Beads were washed twice with wash buffer, resuspended in 400µl of RIP immunoprecipitation buffer (20 mM EDTA pH 8.0 and 40 U mL^{-1} RNase inhibitor in wash buffer) and incubated with 500µl of cell lysate. Part of the cell lysate was used as Input for RNA isolation and SDS-PAGE. Beads were incubated with rotation for 10 h at 4°C. Next, beads were washed three times with wash buffer. A ninth of the bead fraction was used for SDS-PAGE, the rest was further processed for RNA isolation using the miRNeasy mini kit (Qiagen). Twenty nanograms of RNA were pre-amplified using the Ovation PicoSL WTA System V2 (NuGEN) to increase the efficiency of the detection without affecting differences between treatments. The expression of lncWDR59 was analyzed by qPCR and amplification products run on 2% agarose gel. Data were analyzed and represented using the formula: ΔCTAgo2-IP=CTinput-CTAgo2-IP; ΔCTIgG-IP=CTinput-CTIgG-IP; ΔΔCT=ΔCTAgo2-IP-ΔCTIgG-IP; and FE=2ΔΔCT.

Efficiency of Numb-IP and Notch1 (NICD) binding to Numb was assessed by SDS-PAGE. Briefly, protein concentration was measured with Lowry protein assay. Thirty µg of Input and 20µl of IP protein samples were used. Samples were eluted in SDS-PAGE loading buffer, beads separated on a magnet and suspensions electrophoresed on NuPAGE Novex precast gels of 4-15% range (Invitrogen). Proteins were blotted on 0.2µm polyvinylidene fluoride (PVDF) membrane, blocked for 1 h in 10% milk PBST (0.1% Tween-20 in PBS) and incubated with anti-Numb (0.25 µg ml^{-1} in 5% milk) or anti-activated Notch1 (156 µg ml^{-1} in 5% milk) antibodies overnight at 4°C. Membranes were washed three times in PBST and incubated with HRP-conjugated secondary antibody. Membranes were washed three times with PBST and proteins detected using SuperSignal West Pico Chemioluminescent Substrate (Thermo fisher Scientific), followed by analysis with the CCD camera detection system Las4000 Image Quant (GE Healthcare).

Supplementary Figures

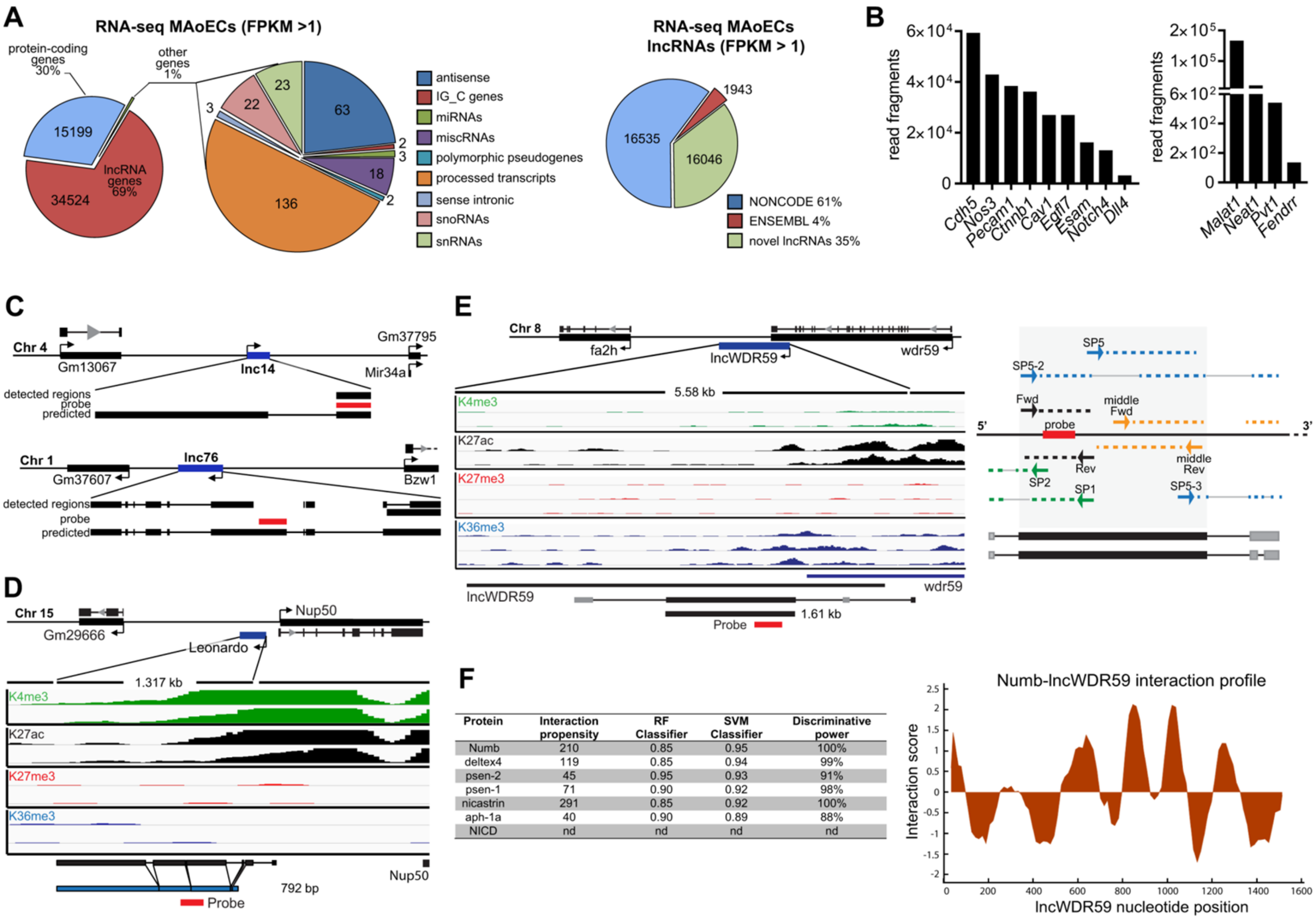

Protein	Interaction propensity	RF Classifier	SVM Classifier	Discriminative power
Numb	210	0.85	0.95	100%
deltex4	119	0.85	0.94	99%
psen-2	45	0.95	0.93	91%
psen-1	71	0.90	0.92	98%
nicastrin	291	0.85	0.92	100%
aph-1a	40	0.90	0.89	88%
NICD	nd	nd	nd	nd

Supplementary Figure 1. RNA-seq of MAoECs for lncRNAs sequencing and lncWDR59-protein interaction. (A) Pie graphs of protein-coding and long non-coding RNA genes expressed in MAoECs expression from RNA-seq analysis. **(B)** Absolute expression of endothelial protein-coding and lncRNA genes from RNA-seq of MAoECs. **(C-E)** Chromosome localization of new identified lncRNAs from RNA-seq of MAoECs. **(D-E)** Trimethylation state of histone 3 of lysine 4 (K4m3), lysine 36 (K36m3), lysine 27 (K27me3), and acetylation of lysine 27 (K27ac) identifying putative transcript and promoter regions of Leonardo and lncWDR59. **(D)** Leonardo gene (1.317 kb long), localized on chromosome 15, transcript (792 bp long), and probe (red) from genome-wide microarray. **(E)** (left) LncWDR59 gene (5.58 kb long), localized on chromosome 8, transcript (1.61 kb long), and probe (red) from genome-wide microarray. (right) Schematic representation of primers design for Hot-Start 3`RACE (SP5, SP5-2, SP5-3) and 5´RACE (SP1, SP2) PCR. Middle Forward Reverse (middle Fwd, middle Rev) primers were used to confirm the 3´RACE PCR results. Fwd and Rev, flanking the probe sequence, were used to analyze the expression of lnc-WDR59 in all the experiments. **(F)** (left) Interaction propensity scores between lncWDR59 and Notch signaling-elated proteins calculated using the catRAPID prediction algorithm. (right) Numb-lncWDR59 interaction sites identified using the Global score analysis for long non-coding RNA transcripts from catRAPID prediction algorithm. Cdh5: Cadherin 5, Type 2 (Vascular Endothelium); Nos3: nitric oxide synthase 3 (Endothelial cell); Pecam1: platelet/endothelial cell adhesion molecule 1 (CD31); Ctnnb1: catenin (cadherin-associated protein), beta 1 (β-catenin); Cav1: caveolin 1; Egfl7: multiple epidermal growth factor-like domains protein 7; Esam: endothelial cell adhesion molecule; Notch4: Notch (Drosophila) homolog 4; Dll4: delta-like 4 homolog (Drosophila); Malat1: metastasis associated lung adenocarcinoma transcript 1; Neat1: nuclear enriched abundant transcript 1; Pvt1: plasmacytoma variant translocation 1; Fendrr: FOXF1 adjacent non-coding developmental regulatory RNA; RF: random forests; SVM: support vector machine; psen: presenilin; aph-1α: alphaprotein-1 alpha.

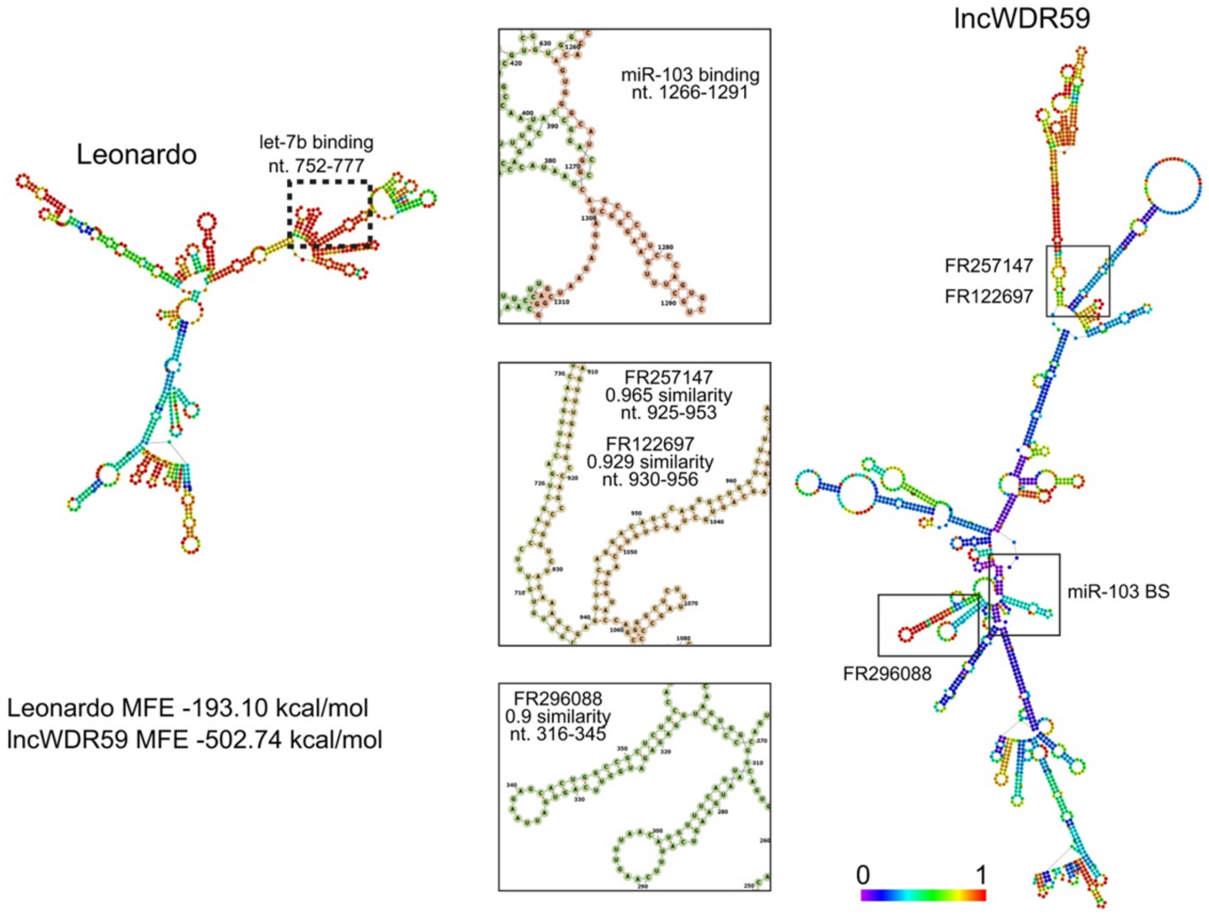

Supplementary Figure 2. Predicted secondary structure of Leonardo and lncWDR59. RNAfold prediction of lncRNA Leonardo and lncWDR59 secondary structure, with a minimum free energy (MFE) of -193 and -502 kcal mol^{-1}, respectively. Colors represent base-pair probabilities. Predicted binding site for let-7b on Leonardo transcript at nucleotide 752-777 and for miR-103 on lncWDR59 transcript at nucleotides 1266-1291 are represented. Functional lncWDR59 sequences showing a similarity ≥ 0.9 with known functional non-protein coding RNA transcript sequences analyzed using the RegRNA 2.0 algorithm are here reported (FR motif IDs).

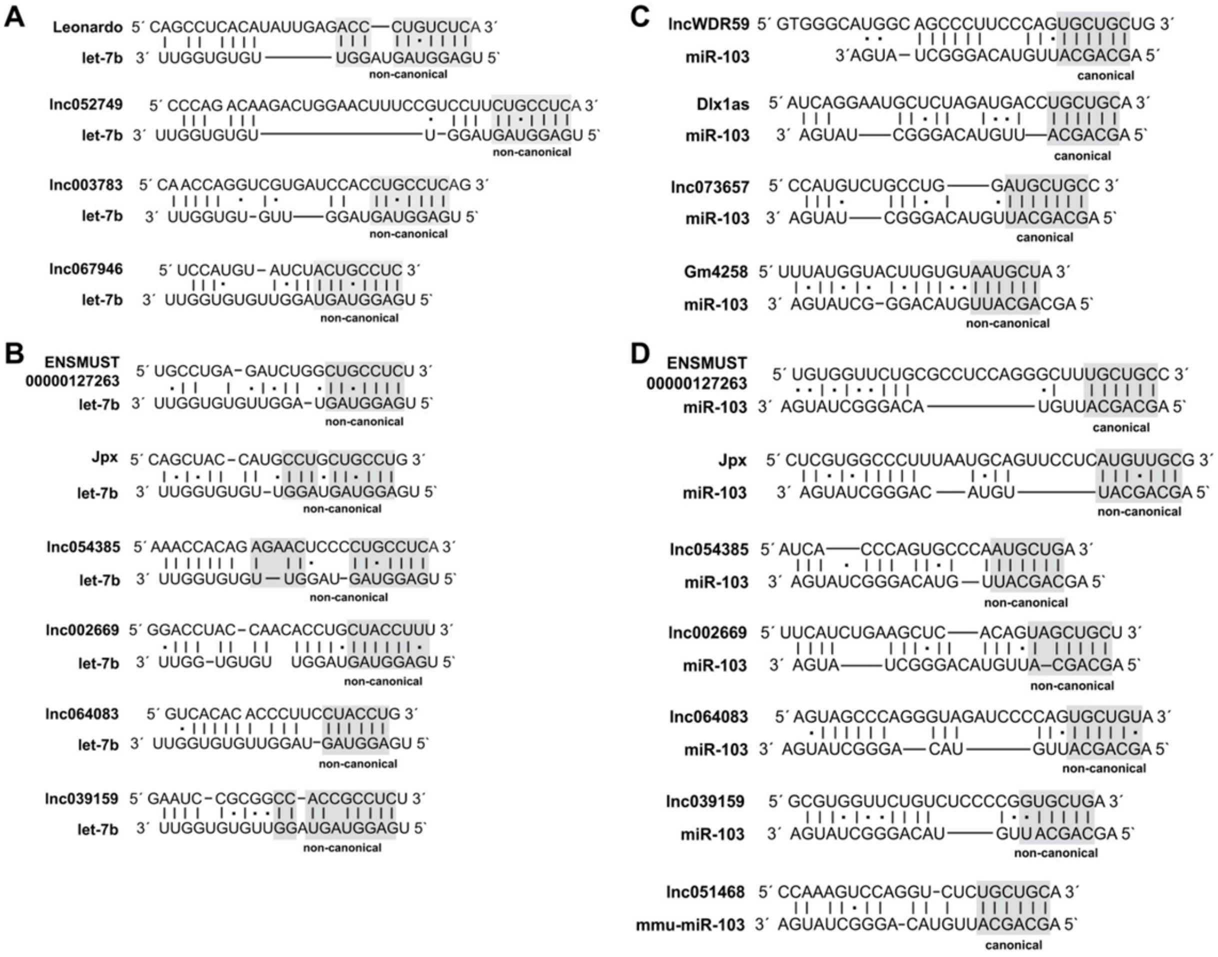

Supplementary Figure 3. Predicted binding site for let-7b and miR-103 on lncRNA transcripts. RNAHypbrid prediction of binding sites for let-7b **(A,B)** and miR-103 **(C,D)** with an energy cut off > -25 on lncRNA transcripts significantly upregulated (FC> 1.5) in EC-Dicer^{flox} mice fed 12 weeks of HFD. LncRNAs containing binding sites specifically for let-7b **(A)**, miR-103 **(C)** or for both miRNAs **(B and D)** were divided in canonical and non-canonical as described by Bartel[18], admitting only 1 G-U mismatch in the seed-target bindings.

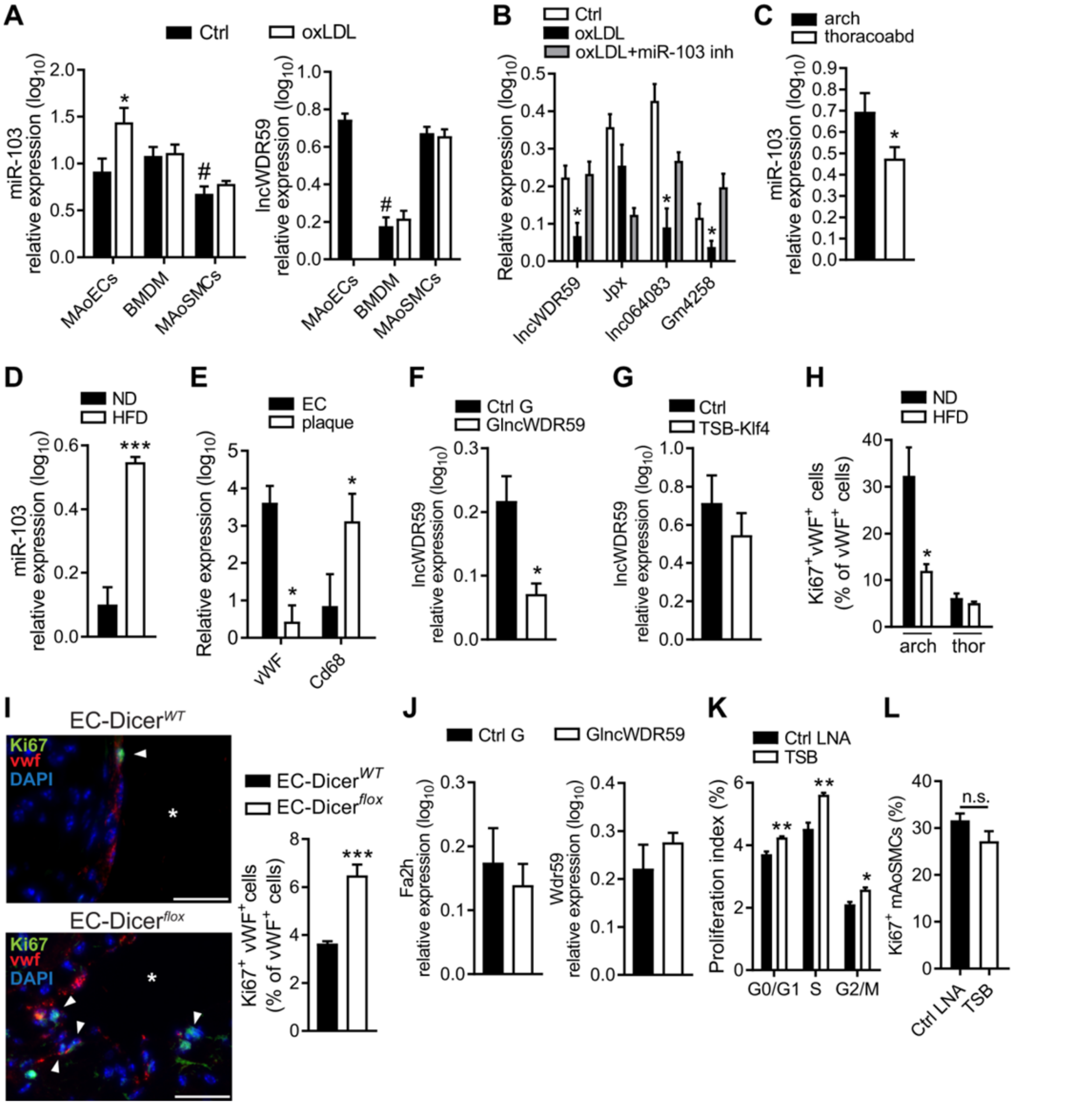

Supplementary Figure 4. miR-103 expression in oxLDL treated MAoECs and EC proliferation during atherosclerosis. (A) miR-103 expression in MAoECs, bone marrow derived macrophages (BMDM) and murine aortic smooth muscle cells (MAoSMCs) treated for 24 h (BMDM for 72 h) with 100µg ml^{-1} oxLDL (n= 6-10 per group). **(B)** Expression of miR-103-predicted lncRNA targets in oxLDL-stimulated MAoECs treated with or without miR-103 inhibitors (50nM) for 24 h (n=4-8 per group). **(C-D)** Expression of miR-103 *in vivo*, in the arches and thoracoabdominal aortas of 12 weeks ND-fed *Apoe*$^{-/-}$ mice **(C)** and in the aortas of 12 weeks ND- or HFD-fed *Apoe*$^{-/-}$ mice **(D)** (n = 3-5 mice per group). **(D)** Expression of endothelial cell (vWF) and macrophages (Cd68) markers in ECs and plaques isolated from *Apoe*$^{-/-}$ mce fed 4 weeks of high fat diet using a laser microdissection system (n= 3-7 mice per group). **(E)** Expression of lnc-WDR59 in *Apoe*$^{-/-}$ mice fed for 8 weeks with HFD, injected during the last 4 weeks of diet with a TSB against the binding of miR-103 on Krüppel-like factor 4 (KLF4). Differences are here considered not statistically significant (n= 4-8 mice per group). **(F-G)** Aortic and thoracic aortae from 12 weeks ND- or HFD-fed mice **(F)** or roots from EC-Dicer^{WT} and EC-Dicer^{flox} HFD-fed mice **(G)** were stained for Ki67 and vWF. DAPI was used to stain the nuclei. Data are expressed as number of Ki67^{+}vWF^{+} cells normalized

on total number of vWF$^+$ cells and expressed in percentage (n= 3-5 mice per group). Stars indicate the lumen. **(H)** MAoECs were treated for 24 h with TSBs and stained with propidium iodide to analyze cell cycle phases by flow cytometry (n=4 per group). **(I)** Analysis of Ki67 immunostaining in MAoSMCs transfected for 24 h with TSBs. Data are normalized and expressed as percentage of the total number of cells (n = 4 per group). **(J)** LncWDR59, Fa2h and Wdr59 expression in MAoECs treated for 24 h with lncWDR59 or control gapmers (n= 3-6 per group). B2m and sno135 were used as housekeeping genes and relative expression analysis. oxLDL: oxidized low-density lipoproteins; inh: inhibitors; thoracoabd.: thoraco-abdominal aortae; ND: normal diet; HFD: high-fat diet; EC: endothelial cells; TSB: target site blockers; vWF: von Willebrand Factor; Cd68: cluster of differentiation 68; n.s. statistically not significant. Data are represented as mean ± SEM of the indicated number (n) of repeats. *P<0.05; **P<0.01; ***P<0.001 from *t*-test and two-way ANOVA statistical analysis. Scale bar: 25µm.

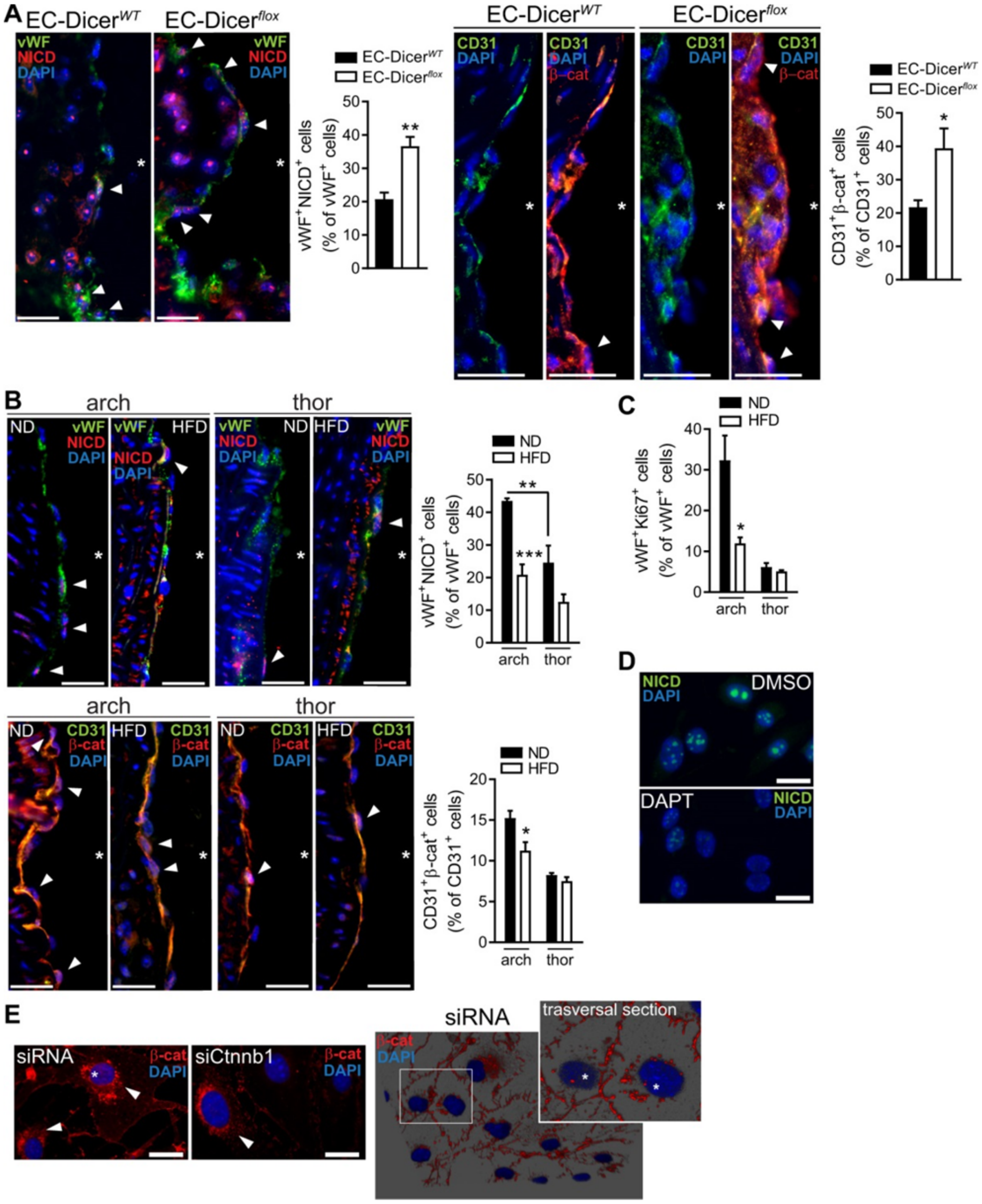

Supplementary Figure 5. Notch1 and β-catenin activation in vivo during atherosclerosis. (A,B) Activated Notch1 (NICD) and β-catenin staining of EC-Dicer^{WT} and EC-Dicer^{flox} roots from mice fed 12 weeks of HFD **(A)**, or of *Apoe*$^{-/-}$ arches and thoracic aortae from mice fed 12 weeks of ND or HFD **(B)**. Quantification of nuclear NICD and β-catenin (β-cat) has been performed by counting the number of NICD/vWF or β-catenin/CD31 double positive cells normalized on total number of vWF or CD31 positive cells and expressed in percentage (n= 3-5 mice per group). **(C)** Quantification of Ki67^{+}vWF^{+} cells normalized on total number of vWF^{+} cells, in percentage, from *Apoe*$^{-/-}$ arches and thoracic aortae of mice fed 12 weeks of ND or HFD (n= 3-5 mice per group). **(D-E)** NICD **(D)** or β-catenin staining **(E)** in MAoECs treated with DAPT or Ctnnb1 siRNAs for 24 h to test the efficiency of Notch and β-catenin inhibition, respectively (n= 4 per group). Inhibition of β-catenin activation makes β-catenin visible only as membrane adaptor protein for cell-to-cell intercellular adhesions. Representative pictures of β-catenin nuclear and perinuclear localization (trasversal section) acquired with confocal microscope. DAPI was used to stain

the nuclei. ND: normal diet; HFD: high-fat diet; EC: endothelial cells; thor: thoracic aorta; NICD: Notch intracellular domain; vWF: von Willebrand Factor; platelet/endothelial cell adhesion molecule 1 (CD31); DMSO: Dimethyl sulfoxide; DAPT: γ-secretase inhibitor; siRNA: small interfering RNA; Ctnnb1: Catenin beta 1. Data are represented as mean ± SEM of the indicated number (n) of repeats. *P< 0.05 from *t*-test and two-way ANOVA statistical analysis. Scale bar: 25μm.

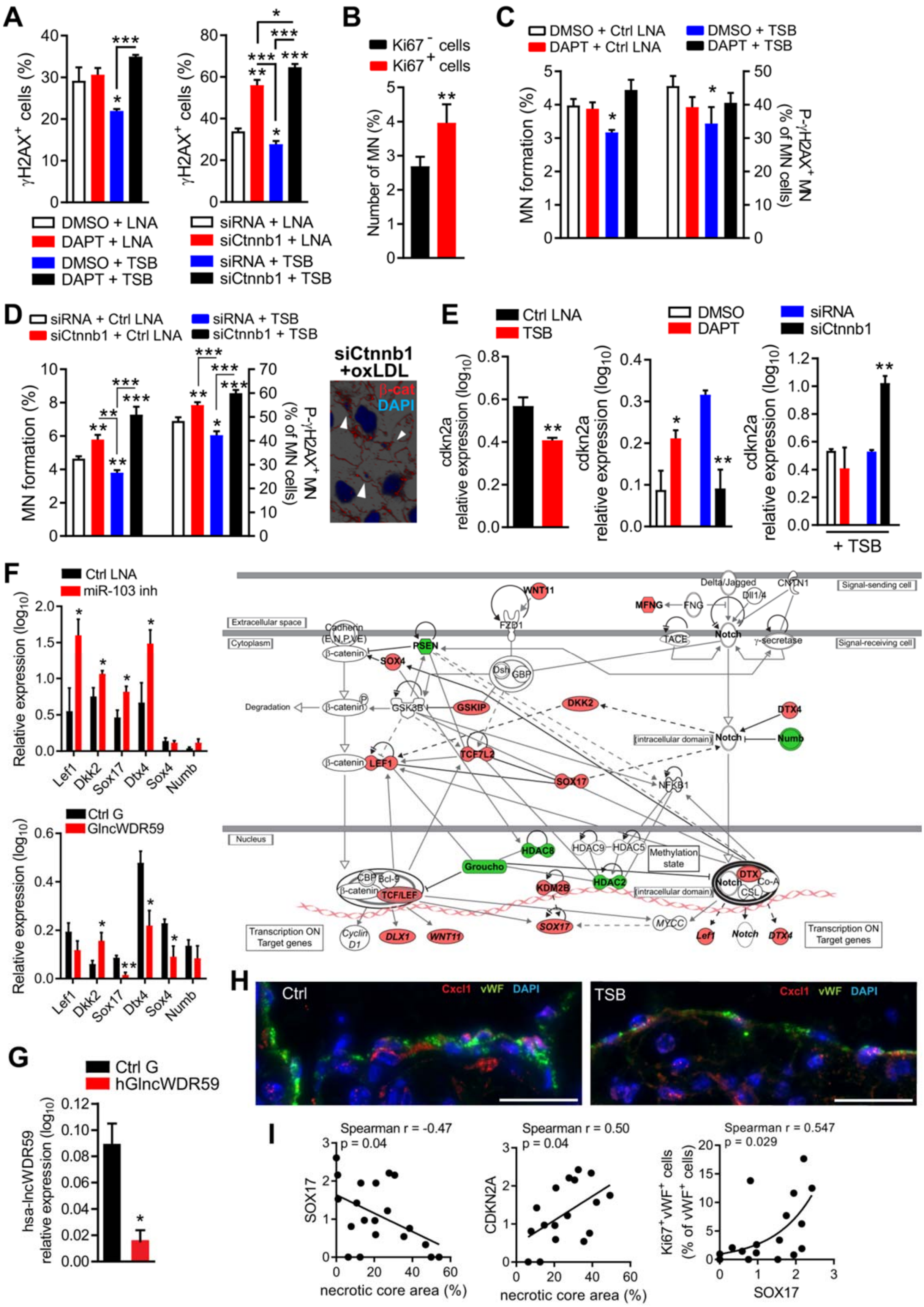

Supplementary Figure 6. Role of lncWDR59 in the regulation of Wnt and Notch1 signaling pathways, DNA damage and MN formation. (A) γH2AX staining in MAoECs treated for 24 h with DAPT inhibitors or Ctnnb1 siRNAs, with or without TSB (n= 4-8 per

group). **(B)** MN formation in proliferating and non proliferating MAoECs, following Ki67 staining. Pair test analysis (n= 7 per group). **(C,D)** MN formation and γH2AX$^+$ MN MAoECs treated with DAPT **(D)** or siCtnnb1 **(E)**, alone or in combination with TSB for 24 h. In panel **D**, a representative picture of MN localization in MAoECs treated with oxLDL and Ctnnb1 siRNAs for 24 h, stained for nulcei (DAPI) and β-catenin. **(E)** Expression of cdkn2a in MAoECs treated for 24 h with TSB, alone or in combination with DAPT inhibitor or Ctnnb1 siRNAs. B2M was used as housekeeping gene and relative expression analysis (n= 4-8 per group). **(F)** Ingenuity pathway analysis representation of Wnt and Notch1 signaling pathways genes, up- (red) or down- (green) regulated in the aorta from EC-Dicer^{flox} mice compared to EC-Dicer^{WT} mice fed 12 weeks of HFD (n= 2 mice per group). The expression of Lef1, Dkk2, Sox17, Dtx4, Sox4 and Numb was further analyzed in MAoECs treatd for 24 h with miR-103 or lncWDR59 inhibitors by qPCR. B2M was used as housekeeping gene and relative expression analysis (n= 5 per group). **(G)** Expression of hsa-lncWDR59 in HAoECs treated for 48 h with hsa-lncWDR59 inhibitors (hGlncWDR59). GAPDH was used as housekeeping gene and relative expression analysis (n= 6 per group). **(H)** Immunofluorescence staining of Cxcl1 on 12 weeks HFD-fed Apoe*Apoe*$^{-/-}$ mice tail vein injected with TSB or control LNAs (aortic roots). vWF and DAPI were used to stain ECs and nuclei, respectively (n = 5 mice per group). **(I)** Correlation of the relative expression levels of SOX17 and CDKN2A in numan carotid lesions with necrotic core area and Ki67 endothelial staining (Ki67$^+$vWF$^+$) (n= 17-21 per group). ND: normal diet; HFD: high fat diet; thor: thoracic aorta; DMSO: Dimethyl sulfoxide; DAPT: γ-secretase inhibitor; siRNA: small interfering RNA; Ctnnb1: Catenin beta 1; MN: micronuclei; γH2AX$^+$ MN: γH2AX positive micronuclei from micronucleated cells; cdkn2a: cyclin dependent kinase inhibitor 2a. Data are represented as mean ± SEM of the indicated number (n) of repeats. *P< 0.05; **P<0.01 from *t*-test and one-way ANOVA statistical analysis. Scale bar: 25µm.

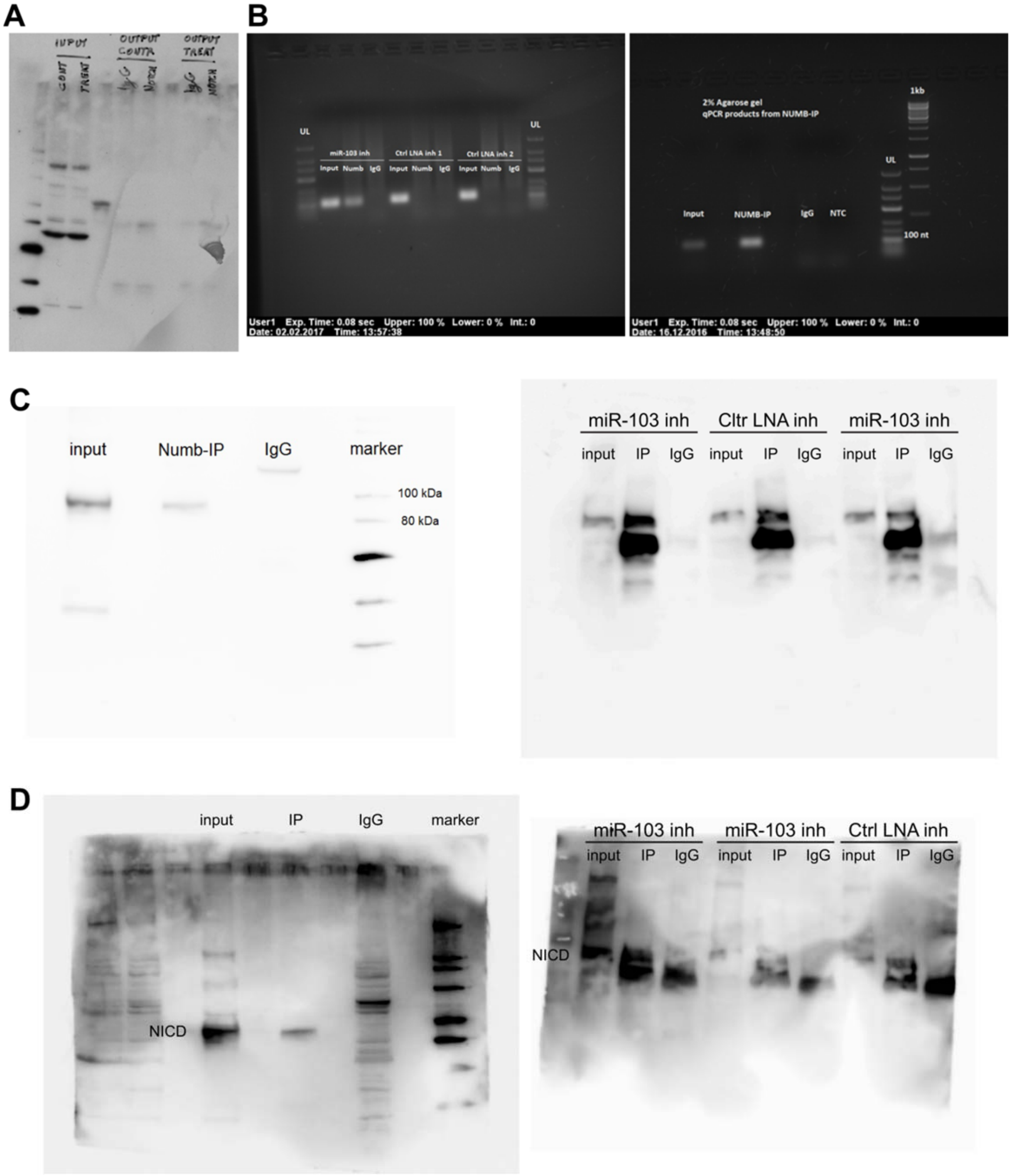

Supplementary Figure 7. qPCR and Western Blot from NICD and Numb-IP. (A) Representative western blot to confirm the immunoprecipitation (IP) of activated Notch1 (NICD, 80 kDa) after treatment of MAoECs with control LNA-inhibitors (contr) or miR-103 inhibitors (Treat). IgG were used as negative control (n = 3 independent experiments per group). (B) Representative agarose gels loaded with the amplification products from lncWDR59 amplifications after qPCR. MAoECs were transfected with miR-103 or control inhibitors for 24 h and Numb-IP was performed (n = 3 independent experiments per group). **(C,D)** Representative western blot for protein Numb (80 kDa) **(C)** to confirm the efficiency of Numb-IP, and for NICD **(D)** in MAoECs treated for 24 h with miR-103 or control LNA inhibitors (n = 3 independent experiments per group). UL: Ultra low Range DNA-Leiter II perGOLD (732-3300, Peqlab). 1kb: DNA high molecular weight marker).

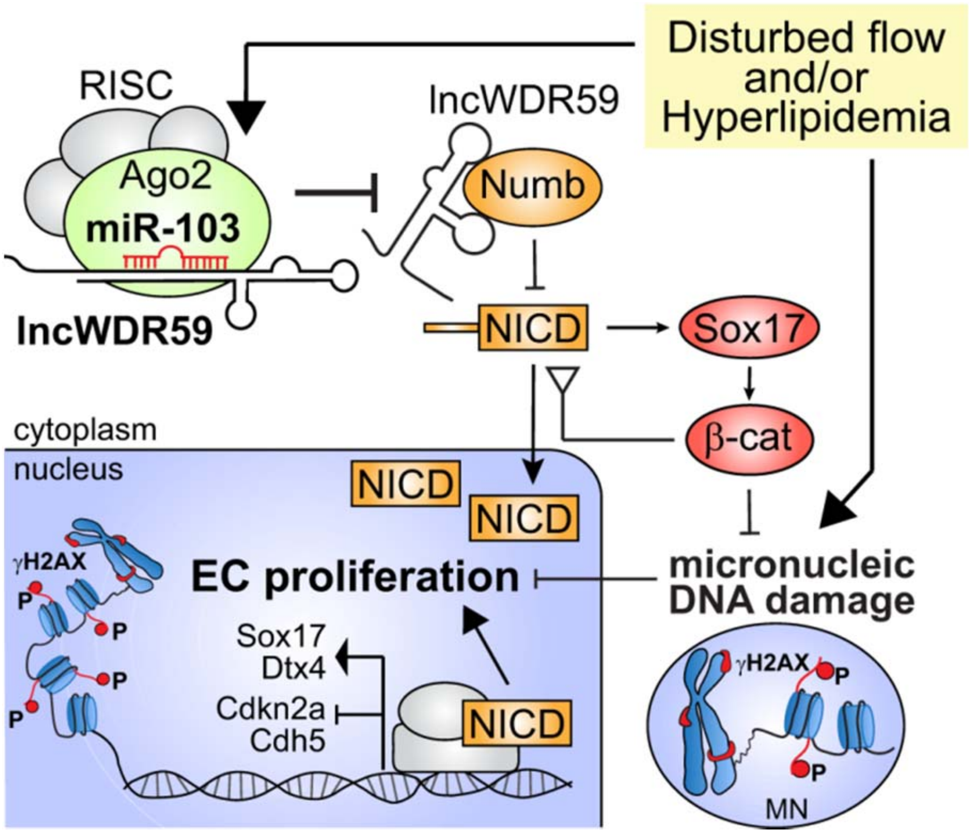

Supplementary Figure 8. LncWDR59-mediated endothelial adaptation. MiR-103 targets lncWDR59 transcript in the RISC of endothelial cells to impair endothelial proliferation and to promote micronucleic DNA damage accumulation. MiR-103 impairs Notch1 activation by promoting Numb interaction with Notch1 (NICD). LncWDR59 promotes NICD transcriptional activity and EC proliferation by targeting Notch1-inhibitor Numb and competing for NICD interaction and degradation. LncWDR59-mediated NICD activation enhances EC proliferation through upregulation of proliferating genes (e.g. Dtx4, Sox17) and inhibition of senescence-associated genes (e.g. Cdkn2a, Cdh5). To protect proliferating cells from mitotic aberrations, lncWDR59 limits NICD activity through Sox17-mediated β-catenin activity, which prevents aberrant proliferation and associated micronuclei formation, where DNA damage accumulates and affects EC function. Hyperlipidemia and oxLDL enhance miR-103 expression, therefore block the protective effect of lncWDR59 on EC maladaptation by inhibiting endothelial regeneration and increasing micronucleic DNA damage accumulation. White up-side-down arrowhead indicates that β-catenin limits Notch1 activity. RISC: RNA-induced silencing complex; Ago2: Argonaute 2; NICD: Notch1 intracellular domain; β-cat: β-catenin; MN: micronucleus; EC: endothelial cell.

Supplementary Tables

Supplementary Table 1. PCR primer sequences

5’ RACE PCR (5´-3´)

SP1	TTAGCTTCACGGTGGGGTAG
SP2	CTGTTGTGGGACCAGCATC
PCR anchor primer	GACCACGCGTATCGATGTCGAC

3’ RACE PCR (5´-3´)

SP5	TAATGGAAGCCCTGTCCCTG
SP5-2	GCCGCAGGTATCTGAGTGTATT
SP5-3	TGCAGGCTTTGTACCCCATA
middle Fwd	CAGACGTTTGTAACTCCCATTC
middle Rev	GTGAGCACCAAGTAGTGGGC

qPCR (5´-3´)

	Forward	Reverse
lncWDR59	GCCGCAGGTATCTGAGTGTATT	CTGTTGTGGGACCAGCATC
lnc052749	GCTGGAGATGTGAGGCAGTT	AAAGCAGTGTGGGCACCTAAG
lnc064083	GGAGTAGCCGAAACCCGAAT	AACCGAGAATCGACAAGCCA
Jpx	ATCACCTCCCCAGTGCTTCG	CCAGGCTCCTCATAGAAATGGCT
Leonardo	AAGTACTCCAGAGATAGGGCA	AGGATCTTCTTGTTACTCTGGCT
Malat1	TGTGACGCGACTGGAGTATG	GGACTCGGCTCCAATCACAA
lnc067946	AGTACCCATTCCTTCTGCCC	GCTATCCCAGTCTAGCATTGCC
lnc039159	TCCCACAAGAAGGAAGAGTAACC	GGTGCAATCCCACACTACCA
lnc073657	GTTGCCTAGTTACTGCCCAGAG	TGACGAAGATTGCCAGAGCG
lnc054385	TCAGCGTCTCTAATGCAGGC	CCGTTCTCCAAGAGTCAGGC
lnc002669	CGTGTGTGTTCCCTAACCCC	TGAACTGGATGCTATGGTGCT

lnc051468	CCTCTCTGACTTGGTTGTCTGTGT	ACCCTCTAGGTGGGCTGCT
lnc064083	GGAGTAGCCGAAACCCGAAT	AACCGAGAATCGACAAGCCA
lnc012953	TGGTTAGCAGGGCACGTTTC	TCTGTCCTTGGGCTTGTCAT
	Forward	Reverse
Dlx1as	GTCCCCTCGATGTGTTCCC	ATTGCCTTCGTCACCGTAGTT
Gm4258	GAGATTCAGCTTGGCCCTTT	TCATTCACAGTAGCCCTGAGGT
ENSMUST00127263	CGCTGGATGGACAGTGGGGT	GCTGGGAGATGGAAAGACAAC
Klf4	GACTAACCGTTGGCGTGAGG	CGGGTTGTTACTGCTGCAAG
Cd68	TTGGGAACTACACGTGGGC	CGGATTTGAATTTGGGCTTG
Ccl2	CTGTAGTTTTTGTCACCAAGCTCAA	AGACCTTAGGGCAGATGCAG
Nos3	AGGCAATCTTCGTTCAGCCA	TAGCCCGCATAGCGTATCAG
Cdkn2a	CGAGGACCCCACTACCTTCT	TCTTGATGTCCCCGCTCTTG
Lef1	GATCCTGGGCAGAAGATGGC	GCTGTCTCTCTTTCCGTGCT
Dkk2	TCTAGGAAGGCCACACTCCA	TGGGTCTCCTTCATGTCCTTT
Sox17	TTCCATCTCCACCTCCGACC	GTCGATTGGCACCTTTCACC
Dtx4	TGTGCCTGTGAAAAACTTGAATG	TGGGATGGACTTTATCTCACTCT
Sox4	GACATGCACAACGCCGAGAT	TTGCCCGACTTCACCTTCTTTC
Numb	CAGTCTTCTGGTGCTGCCTCTC	CCGCACACTCTTTGACACTTCTT
Fa2h	CACACAAGGGCTCCTACCTG	GTGCTGATGCCAAACCCTGA
Wdr59	AACAACGCTGCTCCTTCCTTC	CAGCTCTGGTTGCGTTTCAC
b2m	TCGGTGACCCTGGTCTTTCT	TTTGAGGGGTTTTCTGGATAGCA
Gapdh	CATGGCCTTCCGTGTTCCTA	CCTGCTTCACCACCTTCTTGAT
hsa-lncWDR59	GAGCCCAGCACTAAACCTCT	GCTTATACCTCGTCCCCTGT
SOX17	CCAAGGGCGAGTCCCGTATC	CACGACTTGCCCAGCATCTTG
CDKN2A	TCCCCGATTGAAAGAACCAGA	CTGTAGGACCTTCGGTGACTGA
GAPDH	AGGGCTGCTTTTAACTCTGGT	CCCCACTTGATTTTGGAGGGA

Supplementary Table 2. Upregulated lncRNAs in 12 weeks HFD-fed EC-Dicer^{flox} mice and their expression in Lys-Dicer^{flox} mice

	EC-Dicer^{flox}/ EC-Dicer^{WT}		Lys-Dicer^{flox}/ Lys-Dicer^{WT}						
Probe	p value	FC	p value	FC	Description	Strand	Gene symbol	Annotation	RNA-seq
P01030345	0.0001	2.80	0.4550	-1.23	lincRNA:chr8:113968152-113973727	-	lncWDR59		x
P01023671	0.0003	2.92	0.6839	-1.17	lincRNA:chr5:22058025-22072475	+	6030443J06Rik	ENSMUSG00000097207	x
P01030264	0.0003	2.07	0.3328	1.52	lincRNA:chr6:90915489-90940052	+			
P01027965	0.0003	2.12	0.3375	-1.37	lincRNA:chr11:117835849-117842574	+		NONMMUT012953	x
P01019079	0.0004	2.11	0.4357	-1.07	lincRNA:chr15:102100397-102105872	+	Six3os	NONMMUT025109	x
P01026906	0.0006	1.57	0.6942	-1.15	lincRNA:chr12:70213282-70225157	-			
P01022234	0.0010	2.35	0.3979	-1.33	lincRNA:chr7:144563845-144603732	+		NONMMUT064083	x
P01025047	0.0010	1.80	0.1351	-2.04	lincRNA:chrX:165792277-165807952	-			
P01029644	0.0016	2.34	0.7092	-1.33	lincRNA:chr6:100162756-100178531	-			
P01020932	0.0019	1.57	0.5810	1.25	lincRNA:chr16:21794415-21815365	+		NONMMUT025900	x
P01020004	0.0019	2.50	0.5685	-1.16	lincRNA:chr4:133212951-133214017	-		NONMMUT049938	x
P01025285	0.0020	3.01	0.6400	1.15	lincRNA:chr8:59966759-59989065	-			
P01029005	0.0021	1.57	0.3727	-2.02	lincRNA:chr5:14943275-14944205	+			
P01022422	0.0024	1.92	0.4072	1.34	lincRNA:chr4:149383134-149451259	+	Lnc14		x
P01027182	0.0027	1.54	0.1877	1.34	lincRNA:chr15:62090565-62091770	+		NONMMUT023726	x
P01019922	0.0027	1.88	0.5030	1.12	lincRNA:chr5:74487385-74497428	-		NONMMUT052749	x
P01029502	0.0030	2.50	0.0652	1.58	lincRNA:chr5:52461266-52541416	-	lnc PPARGC1A	NONMMUT052270	x
P01025199	0.0035	2.99	0.7791	1.16	lincRNA:chr8:126268510-126290810	+			
P01020791	0.0038	3.09	0.9716	1.02	lincRNA:chrX:100676170-100710920	+	Jpx	NONMMUT073474	x
P01029597	0.0039	2.09	0.0640	-2.10	lincRNA:chr2:174428737-174439062	-		NONMMUT041655	x
P01033597	0.0040	1.51	0.1673	-1.28	lincRNA:chr7:140081171-140123058	-		NONMMUT063965	x
P01029862	0.0043	1.50	0.1521	1.64	lincRNA:chr5:120269342-120279542	-			
P01029543	0.0043	2.48	0.7705	-1.14	lincRNA:chr2:113880070-113887230	+		NONMMUT039159	x
**P01024649**	**0.0045**	**1.55**	**0.0012**	**-1.54**	**lincRNA:chr9:96964067-96973567**	**-**			
P01024616	0.0056	1.60	0.1903	-1.55	lincRNA:chr2:72818943-72826893	+		NONMMUT037893	x
P01022349	0.0061	4.31	0.1756	-1.66	lincRNA:chr1:139806645-139809498	+	Gm4258	NONMMUT002921	x
P01022950	0.0061	1.77	0.1483	-2.26	lincRNA:chr5:14923900-15062725	+			
P01030056	0.0065	2.25	0.1156	1.35	lincRNA:chr11:51854060-51903278	+			
P01022216	0.0067	2.22	0.3483	-1.30	lincRNA:chr1:139795171-139796306	+		NONMMUT002916	x
**P01026309**	**0.0070**	**1.62**	**0.0327**	**-1.43**	**lincRNA:chr8:91569369-91594119**	**+**			
P01021087	0.0072	2.57	0.1716	1.17	lincRNA:chr1:64686823-64728598	+			
P01031603	0.0074	2.03	0.9036	1.06	lincRNA:chr11:112905201-113062945	-		ENSMUST00000127263	x
P01026452	0.0083	2.07	0.2728	-1.76	lincRNA:chr1:173254292-173257445	-		NONMMUT003783	x
P01018882	0.0089	1.99	0.8417	1.08	lincRNA:chr1:74114371-74115267	-			
P01031835	0.0091	2.75	0.1563	1.53	lincRNA:chr5:149801336-149806799	-			
**P01022508**	**0.0092**	**1.67**	**0.0424**	**-1.39**	**lincRNA:chr5:54053882-54054174**	**+**			
**P01019457**	**0.0119**	**3.57**	**0.0313**	**-1.53**	**lincRNA:chr15:73455424-73477275**	**-**			
**P01028116**	**0.0120**	**1.71**	**0.0031**	**2.89**	**lincRNA:chr17:15100982-15115630**	**-**			
P01022975	0.0125	2.60	0.1376	1.38	lincRNA:chr1:90149027-90152254	+			
P01021706	0.0137	1.78	0.0764	-1.64	lincRNA:chr6:100518251-100519726	-		NONMMUT058237	x
P01022502	0.0141	2.28	0.1945	-1.55	lincRNA:chr4:124127144-124334840	-		NONMMUT049357	x
P01029833	0.0149	1.93	0.3450	1.29	lincRNA:chr14:78924115-78938125	-			

P01032997	0.0153	1.51	0.6036	-1.59	lincRNA:chr1:85117750-85129200	-			
P01025061	0.0158	1.80	0.0824	-1.76	lincRNA:chr10:126818382-126856582	-			
P01018299	0.0168	2.25	0.0793	-1.88	lincRNA:chr15:10363330-10371830	+			
P01026891	0.0170	1.58	0.7217	-1.11	lincRNA:chr13:107812547-107823601	+		NONMMUT019054	x
P01019218	0.0170	1.62	0.3403	1.25	lincRNA:chr5:107503800-107515575	-		NONMMUT053561	x
P01033151	0.0177	2.42	0.6579	1.11	lincRNA:chr5:100845342-100869667	-			
P01022142	0.0177	2.18	0.3749	-1.38	lincRNA:chr4:21683562-21694344	-			
P01030269	0.0181	1.67	0.7557	-1.24	lincRNA:chr11:95657291-95657772	-			
P01028465	0.0183	2.20	0.1735	1.92	lincRNA:chr1:135956616-135957485	-		NONMMUT002669	x
**P01026373**	**0.0201**	**2.35**	**0.0413**	**-1.43**	**lincRNA:chr14:26973425-26986650**	**-**			
P01024624	0.0210	1.58	0.0787	-2.77	lincRNA:chr12:110002978-110003360	-		NONMMUT015662	x
P01028929	0.0212	1.50	0.0579	-3.04	lincRNA:chr1:58440355-58449627	-		NONMMUT001025	x
P01027427	0.0215	1.78	0.2120	-1.84	lincRNA:chr4:123572090-123581365	+			
P01030246	0.0216	1.73	0.5130	1.17	lincRNA:chr4:123572090-123581365	-			
P01026576	0.0223	1.65	0.5110	-1.14	lincRNA:chr2:153643200-153654975	-			
P01028722	0.0243	2.58	0.8619	-1.05	lincRNA:chr2:153163620-153170845	-		NONMMUT040572	x
P01032123	0.0247	1.89	0.6465	1.14	lincRNA:chr8:69416817-69417583	+		NONMMUT065850	x
P01024172	0.0259	2.78	0.2344	-1.67	lincRNA:chr2:84422868-84429877	+			
**P01030200**	**0.0261**	**5.42**	**0.0300**	**1.87**	**lincRNA:chr8:124355710-124401160**	**+**			
P01028043	0.0265	3.08	0.9431	-1.04	lincRNA:chr4:3009566-3009932	-			
P01028507	0.0267	3.18	0.6544	1.28	lincRNA:chr7:71231097-71233680	-			
P01018985	0.0282	2.09	0.2259	1.44	lincRNA:chr7:26054813-26063725	-			
P01025352	0.0283	1.71	0.4856	-1.47	lincRNA:chr17:22013350-22095775	-			
P01021034	0.0283	1.52	0.1238	1.61	lincRNA:chr11:97505439-97522274	+			
P01029551	0.0294	1.68	0.4711	-1.28	lincRNA:chr15:61984389-62102500	+			
P01031426	0.0300	1.88	0.1369	1.72	lincRNA:chrX:102232535-102265435	-		ENSMUST00000127533	x
P01022690	0.0303	1.81	0.6161	-1.20	lincRNA:chr6:112552041-112560067	-			
P01023289	0.0305	1.82	0.6059	-1.18	lincRNA:chr12:81217572-81233910	+		ENSMUST00000180643	x
P01020560	0.0305	1.53	0.5042	1.26	lincRNA:chr9:65376063-65390480	+			
P01018944	0.0314	2.71	0.8734	1.07	lincRNA:chr9:7214754-7225700	+		NONMMUT067946	x
P01029041	0.0317	1.57	0.4057	-1.18	lincRNA:chr19:4210704-4211111	-			
P01031764	0.0328	1.91	0.2962	1.78	lincRNA:chr10:69669025-69686275	+			
P01026653	0.0346	2.14	0.4759	-1.36	lincRNA:chr17:15226181-15250333	+		NONMMUT028352	x
P01030561	0.0358	1.97	0.8659	1.08	lincRNA:chr1:58443698-58449898	-	lnc76		x
P01032293	0.0365	2.33	0.2275	1.64	lincRNA:chr14:77511189-77522852	+		ENSMUST00000137110	x
P01026623	0.0374	2.17	0.3814	-1.85	lincRNA:chr9:40268246-40268441	-			
**P01018653**	**0.0389**	**2.42**	**0.0171**	**2.00**	**lincRNA:chr5:26323150-26357875**	**+**			
P01021774	0.0393	2.25	0.0968	1.73	lincRNA:chrX:106029816-106082235	+		NONMMUT073657	x
P01020121	0.0395	2.24	0.0585	-1.89	lincRNA:chr5:123439974-123448354	+		NONMMUT054385	x
P01032900	0.0401	3.47	0.5356	1.17	lincRNA:chr15:12119755-12127230	+			
P01026110	0.0404	2.29	0.4825	1.28	lincRNA:chr4:53312709-53355184	+			
P01022924	0.0432	1.65	0.3452	-1.57	lincRNA:chr10:66559716-66647841	-			
P01029305	0.0437	1.50	0.4712	-1.28	lincRNA:chr5:22887983-22939658	-		NONMMUT051468	x
**P01026279**	**0.0437**	**2.21**	**0.0209**	**-1.82**	**lincRNA:chr10:39383665-39442690**	**-**			
P01026562	0.0441	1.84	0.3612	-1.48	lincRNA:chr3:41143423-41359623	+			

P01025348	0.0443	2.03	0.5610	-1.45	lincRNA:chr2:129416848-129417413	-			
P01025205	0.0450	1.87	0.2482	-1.43	lincRNA:chr2:174852887-174853961	+			
P01028919	0.0453	1.79	0.7254	1.02	lincRNA:chr6:129151512-129208737	-			
P01027296	0.0474	1.56	0.3610	-1.19	lincRNA:chr15:84737071-84753414	-			
P01018413	0.0481	1.83	0.5479	1.18	lincRNA:chr17:33995525-34028025	-	Dlx1as	NONMMUT029220	x
**P01017666**	**0.0482**	**2.20**	**0.0045**	**1.77**	**lincRNA:chr18:84760623-84761084**	**+**			
P01024974	0.0484	2.16	0.5494	1.25	lincRNA:chr15:84752337-84752984	-	Leonardo		x
P01030427	0.0487	3.29	0.0531	-1.67	lincRNA:chr4:119967625-119978325	+	Foxo6os	NONMMUT049230	x
P01022821	0.0500	2.19	0.4551	1.33	lincRNA:chr7:91558475-91733625	+		ENSMUST00000180387	x
**P01020444**	**0.0508**	**1.84**	**0.0380**	**2.25**	**lincRNA:chr14:65720392-65769859**	**-**			

Supplementary Table 3. Characteristic functional loops identified on lncWDR59 transcript sequence using RegRNA 2.0

Motif Name	Position	Length	similarity	Sequence	Structure
FR048278/Eukaryotic_type_signal_recognition_particle_(SRP)	851 ~ 961	111	0,352518	gggcatggtggcacacgcctctaatccgcatttgggaggcagaggcaggaggatctctgagtttgaggccagcctggtctacaaagcgagttccaggacagccagggctgt	
FR136328/Eukaryotic_type_signal_recognition_particle_(SRP)	864 ~ 961	98	0,3186813	cacgcctctaatccgcatttgggaggcagaggcaggaggatctctgagtttgaggccagcctggtctacaaagcgagttccaggacagccagggctgt	
FR089002/non-protein_coding_(noncoding)_transcript	856 ~ 957	102	0,2769231	tggtggcacacgcctctaatccgcatttgggaggcagaggcaggaggatctctgagtttgaggccagcctggtctacaaagcgagttccaggacagccaggg	
7016_long_fRNAdb_seq.fas	884 ~ 959	76	0,5348837	gggaggcagaggcaggaggatctctgagtttgaggccagcctggtctacaaagcgagttccaggacagccagggct	
FR110345/Eukaryotic_type_signal_recognition_particle_(SRP)	909 ~ 959	51	0,1517028	gagtttgaggccagcctggtctacaaagcgagttccaggacagccagggct	
FR127129/non-protein_coding_(noncoding)_transcript	886 ~ 946	61	0,1120163	gaggcagaggcaggaggatctctgagtttgaggccagcctggtctacaaagcgagttccag	
FR400742/non-protein_coding_(noncoding)_transcript	885 ~ 959	75	0,2700422	ggaggcagaggcaggaggatctctgagtttgaggccagcctggtctacaaagcgagttccaggacagccagggct	
FR382109/4.5S_RNA	885 ~ 933	49	0,4945055	ggaggcagaggcaggaggatctctgagtttgaggccagcctggtctaca	
FR285704/4.5S_RNA	885 ~ 933	49	0,4736842	ggaggcagaggcaggaggatctctgagtttgaggccagcctggtctaca	
FR265345/4.5S_RNA	885 ~ 933	49	0,483871	ggaggcagaggcaggaggatctctgagtttgaggccagcctggtctaca	
FR249718/non-protein_coding_(noncoding)_transcript	885 ~ 933	49	0,4891304	ggaggcagaggcaggaggatctctgagtttgaggccagcctggtctaca	
FR098447/non-protein_coding_(noncoding)_transcript	312 ~ 360	49	0,097561	gggctggagagatggttcagtgattaagagcactggccgctcttccaga	
FR083311/4.5S_RNA	885 ~ 933	49	0,2571429	ggaggcagaggcaggaggatctctgagtttgaggccagcctggtctaca	
FR074601/4.5S_RNA	885 ~ 933	49	0,4891304	ggaggcagaggcaggaggatctctgagtttgaggccagcctggtctaca	
FR012420/4.5S_RNA	885 ~ 933	49	0,4736842	ggaggcagaggcaggaggatctctgagtttgaggccagcctggtctaca	
7062_long_fRNAdb_seq.fas	885 ~ 933	49	0,4945055	ggaggcagaggcaggaggatctctgagtttgaggccagcctggtctaca	
FR072034/non-protein_coding_(noncoding)_transcript	882 ~ 925	44	0,083691	ttgggaggcagaggcaggaggatctctgagtttgaggccagcct	
FR013292/non-protein_coding_(noncoding)_transcript	901 ~ 948	48	0,1369427	ggatctctgagtttgaggccagcctggtctacaaagcgagttccagga	
FR393371/4.5S_RNA	920 ~ 959	40	0,1321429	cagcctggtctacaaagcgagttccaggacagccagggct	

FR297824/small_non-messenger_RNA_(snmRNA)	886 ~ 932	47	0,4574468	gaggcagaggcaggaggatctctgagtttgaggccagcctggtctac	
FR334146/4.5S_RNA	311 ~ 344	34	0,1797753	ggggctggagagatggttcagtgattaagagcac	
FR330524/non-protein_coding_(noncoding)_transcript	885 ~ 933	49	0,4680851	ggaggcagaggcaggaggatctctgagtttgaggccagcctggtctaca	
FR316427/Piwi-interacting_RNA_(piRNA)	930 ~ 958	29	0,9655172	tacaaagcgagttccaggacagccagggc	
FR257147/non-protein_coding_(noncoding)_transcript	925 ~ 953	29	0,9655172	tggtctacaaagcgagttccaggacagcc	
FR188027/4.5S_RNA	885 ~ 933	49	0,4835165	ggaggcagaggcaggaggatctctgagtttgaggccagcctggtctaca	
FR013876/non-protein_coding_(noncoding)_transcript	885 ~ 933	49	0,4631579	ggaggcagaggcaggaggatctctgagtttgaggccagcctggtctaca	
FR220773/transfer_RNA_(tRNA),_GCT_(Ser/S)_Serine	886 ~ 933	48	0,4623656	gaggcagaggcaggaggatctctgagtttgaggccagcctggtctaca	
FR123036/non-protein_coding_(noncoding)_transcript	317 ~ 348	32	0,4225352	ggagagatggttcagtgattaagagcactggc	
FR086575/4.5S_RNA	310 ~ 360	51	0,1480263	gggggctggagagatggttcagtgattaagagcactggccgctcttccaga	
FR378699/Piwi-interacting_RNA_(piRNA)	886 ~ 933	48	0,4479167	gaggcagaggcaggaggatctctgagtttgaggccagcctggtctaca	
FR323422/Piwi-interacting_RNA_(piRNA)	310 ~ 344	35	0,0822622	gggggctggagagatggttcagtgattaagagcac	
FR184934/Piwi-interacting_RNA_(piRNA)	928 ~ 954	27	0,962963	tctacaaagcgagttccaggacagcca	
FR122697/non-protein_coding_(noncoding)_transcript	930 ~ 956	27	0,9285714	tacaaagcgagttccaggacagccagg	
FR122697/non-protein_coding_(noncoding)_transcript	314 ~ 360	47	0,0868644	gctggagagatggttcagtgattaagagcactggccgctcttccaga	
FR102086/Piwi-interacting_RNA_(piRNA)	885 ~ 929	45	0,0826271	ggaggcagaggcaggaggatctctgagtttgaggccagcctggtc	
FR088010/non-protein_coding_(noncoding)_transcript	924 ~ 958	35	0,0692641	ctggtctacaaagcgagttccaggacagccagggc	
FR364545/Piwi-interacting_RNA_(piRNA)	885 ~ 933	49	0,4574468	ggaggcagaggcaggaggatctctgagtttgaggccagcctggtctaca	
FR297251/precursor_micro_RNA_(miRNA)_mir-709	930 ~ 954	25	0,96	tacaaagcgagttccaggacagcca	
FR246311/Piwi-interacting_RNA_(piRNA)	312 ~ 360	49	0,4421053	gggctggagagatggttcagtgattaagagcactggccgctcttccaga	
FR214945/Piwi-interacting_RNA_(piRNA)	888 ~ 916	29	0,9310345	ggcagaggcaggaggatctctgagtttga	

FR189195/non-protein_coding_(noncoding)_transcript	912 ~ 956	45	0,0783133	tttgaggccagcctggtctacaaagcgagttccaggacagccagg	
FR402429/	312 ~ 356	45	0,0866667	gggctggagagatggttcagtgattaagagcactggccgctcttc	
FR250824/Eukaryotic_type_signal_recognition_particle_(SRP)	886 ~ 933	48	0,4516129	gaggcagaggcaggaggatctctgagtttgaggccagcctggtctaca	
FR173288/Piwi-interacting_RNA_(piRNA)	918 ~ 945	28	0,9285714	gccagcctggtctacaaagcgagttcca	
FR118732/non-protein_coding_(noncoding)_transcript	886 ~ 933	48	0,4666667	gaggcagaggcaggaggatctctgagtttgaggccagcctggtctaca	
FR093603/Piwi-interacting_RNA_(piRNA)	887 ~ 922	36	0,0754717	aggcagaggcaggaggatctctgagtttgaggccag	
FR007439/Eukaryotic_type_signal_recognition_particle_(SRP)	886 ~ 933	48	0,4615385	gaggcagaggcaggaggatctctgagtttgaggccagcctggtctaca	
FR263646/Group_I_intron	901 ~ 955	55	0,0951417	ggatctctgagtttgaggccagcctggtctacaaagcgagttccaggacagccag	
FR215728/Group_I_intron	885 ~ 903	19	1	ggaggcagaggcaggagga	
FR128982/Group_I_intron	930 ~ 952	23	0,9565217	tacaaagcgagttccaggacagc	
FR296088/Piwi-interacting_RNA_(piRNA)	316 ~ 345	30	0,9	tggagagatggttcagtgattaagagcact	
FR185909/Piwi-interacting_RNA_(piRNA)	309 ~ 346	38	0,0666667	agggggctggagagatggttcagtgattaagagcactg	
FR335336/Eukaryotic_type_signal_recognition_particle_(SRP)	423 ~ 463	41	0,0853659	atgcctttctctggcctctgtcagcaccagacatgcacatg	
FR312776/BC200_RNA	312 ~ 360	49	0,1322581	gggctggagagatggttcagtgattaagagcactggccgctcttccaga	

Supplementary Table 4. Background information of patients and classification of the human plaque type of lesions

**Gender**	**Percentages in the subjects (nr subjects)**
Male<br>Female	65% (13)<br>35% (7)
**Type of lesions**	**Percentages in the subjects (nr subjects)**
Stable<br>Unstable*	70% (14)<br>30% (6)
**Age (±SD)**	75 (±8,89)
**Risk factors**	**Percentages in the subjects (nr subjects)**
Smoking<br>Hypertension<br>Dyslipidemia<br>Diabetes<br>Obesity<br>Family history of cardiovascular events	25% (5)<br>75% (15)<br>35% (7)<br>25% (5)<br>10% (2)<br>20% (4)

*>30% necrotic core area

Supplementary Notes

Leonardo and lncWDR59 sequence

>lincRNA:chr15:84752337-84752984:-1 (from Ensembl 54: May 2009)

New coordinates lincRNA:chr15: 87056117-87056995:-1 (from Ensembl release 81)

TTCTGCTTGAGTTTTAACGAGAATGTGAACCAGAATGTCAGACCCATCCTTAACCATCCGGAAA
CGGGTCTCAGAGAAACTAGCATTTCTAGAGCAATAGTTCCAAACTTTTGAGAGAGGACAAAGGG
GGTTCCAAGGGAGAAGTCAAGTCCCTTATTTTCAGGGT**GTTTCATCCATCGATCTGCAAGAACTG**
CAAAAGGAGACTAGCTGGCACGGTGGTAGAGTACTTGGCTAGCACGAGGAAAGCCCTAAATTC
AGTTCCCCCAGATCCAGCAGAGTGAAAGAGAAAAAGCCAGATCGCATTTAATGCACACATTTG
TTCTTGATTCAACTGACTGCCAATGAAGTGTCTTCTGAAAATTTGTGTTAAATACATGCAAGGTA
TTTTTCTTGTCATTCTCTACACATTTCAGCTATGTCGTTAACGTCATATTTAGTGCTATAAGTACT
CCAGAGATAATTTAAGTATCTGAAGTCAGTCAGGTCATGGGCA**TTTTCAACTAAAGTCACAGTTA**
CTGGAAGGCTGAGGCAAGAGGATCAGCCCAGGCCAGCCAGAGTAACAAGAAGATCCTCATTA
ATACAGACCAGACCCACAATCACAGCTATTCAGACAGTTGAGGCAGGAGAATCAAAACTTTAA
GGCCTATTCTGGGCCACAGATAAAATTCAAATCTATGTTGAGCGTGCCTGAGCAACTTAGACCC
TCTCCCAAAAATAAATAAAAATACAAAACTGGCTGGTCATAGTGGTGCTCATTTTAATCCCAGT
ACTTGATTAAATCCCAGTACAGAGGCAGATCTCTGAATTCCAGGCTAGCTTGATCTTCATAGTT
CCAAGATGGCCAGCCTCACATATTGAGACCCTGTCTCAAAAGCAAAC

Genomic and transcribed sequence of Leonardo. Genomic sequence of murine lncRNA Leonardo, located on the negative strand of chromosome 15. In bolt, Leonardo transcript sequence (from RNA-seq). Underline: probe sequence; double underline: let-7b predicted binding site.

>lincRNA:chr8:111444252:111449827:-1 (mm9)

ATTTCAGCACTGATACCAGAGTCTCTCTCTGCCTCTGGGCAGGCTGGCTGGGCTCCAG
GGGTACTTTTTACTTGAACTCCCTTGCAAACTCATCATGATTTTCAGGGGTTTCACTTGT
CTATACCAGGGAAGAGAGTTTAAAAAAAAAAACAAGACCCACTATACTGAAATCTCTCTC
TCTTAGAAATTTAAATTCCTTGGGCCTGTCATAAGGCTCAGTGGGCAGAGGTGCCTGCC
ACTAAGCTTACTAACCTCAGTTTTCTCCCTCGCATGAGCCACCATAGAAGAACTGACTCC
TAAGAGTTGTCCTCTGACTTCCACATGTGTGCTTTGATATGTGAATGTACACACACACAT
ACAAAAAATGTAATTTAACAATTTTATGATTCCAAACTAATTAACCGGAGACACATAAGTA
ATTTGTGTCATTGGATTATGGTTGCACGTGTTTGTAGTCCCTTCACTCACGGGGCTGAG
GTAAGAGGGTTTCAAGTTTGAGACCATCCTGGGCTACACAGTGAGGTGATGGCCAGCC
TCACTTATATATGAAGACACTTACAAGCAAACGTCATAAAGAAGATCTGGAGAGATCGCT
TAGTAATCAAATGCTTGGCTAGCATACAGGAGGTGATGTGTTTGATCCTCAGCAACACA
CACACTGACACTCAAACTAAAACAAAACAAAATACTACATGTTCCATAATAGGTAAGTGT
TGTATATCACTTTGTTTGAAACTATGATTTAAGACTCATGAATGGATTAGGAAATCAGTCT
CGTAGATAAAAACCAACACTTTCTATTTTCAAGGATTACTTTGTGTGTGTAAATCTCTGTG
TCTTCAAGTGTATGTTGTGTGGGACAGAAGAGGCCATCTGGTTCCTTGGTGCTAGGATT
ATCAGTCTTTTCAGGACACCTTGTGTCATGTGTGTTCAGGGATCCTAACTTGGATCTTCA
TGATTGCAAAGTCAGAACTGTTTTGTCTCCAGCCCCCAAACTGTCATATAAAGAAAGTAA
AATAATGTGGGTGAGAGACAATCGTGTACTAACACAGTCTGATGTCCTTTGTTTACTAGT
GCGTGCGCACACACACACACACACACACACACACACACACACACACGTACTTATATCTCAT
GGTGGCAGGTGGGCCTCACCTCTCCCCTGTATTACTAATTGGTCATGAGACTCCGTGG
CATAAGCTTGATCTTGAACTATGTGCCAGGTTTGGTACCAGTGCCTCAGCAGCACGTGT
AGTCCGGTGTGCTGGGATGGGGGAGGAAGG**GCCGCAGGTATCTGAGTGTATTACACT**
GTACTCCTCCAGCCTCGTTTGTGTCGACACTGCCGTGATGCTGGTCCCACAACAGGCG
GACTGATGTGCACAGACACAGATCTGTTGGGTCCTCTGTGAGTTCACACCATTTACAG
AGAGCGGTCTCTTCCTCTACCCCACCGTGAAGCTAATGGAAGCCCTGTCCCTGTTTAG
AATGACATAGTTTTCTTGTGTGGATTATTTCTGTAAGAACATTGTCTTCCAAAACAAAA
AAACCTTCTTAGTGACTATGAAGTCATTTCAAGTTTAACATGTTTCATAGGGGGCTGGA
GAGATGGTTCAGTGATTAAGAGCACTGGCCGCTCTTCCAGATGCAAGTCCCAGTAAC
CATAAGCCAGGCCACAGACGTTTGTAACTCCCATTCCGGGGATCAGATGCCTTTCTCT
GGCCTCTGTCAGCACCAGACATGCACATGCTATGTAGACATGCTGGCAAAACACTCTT
TATGCATAAAATAAAAACAAGACACTATGTAACATCTCTCAATAGGTCAGTTCTGTTTG
CATTTCCAAGTTACCAGGACAAAAACTTAGTATTTGTGAGAAATATCCCTGTTTGGAAA
CACAGTGGTCTGGATGCTGGTGGGGTCAGCAGCCTCTCTACCATAGCTAAGTCCTCTT
GGTTTGGTTTACTGTGCTCGTCTGAGGTAGGGCACCGTTGTGTTTCCCAGCTGACCTT
GAACTTGAGACCTCTGACTGCCAGAGTGCTGGGATTCCACCACACCTGGGCTTTATTT
GCTCTTTAAAATATTATAACCTGTGTCCTAAACACCTTTGAATTTGCCAGGGAGAACAC
TAGGCATGGGCATGGTGGCACACGCCTCTAATCCGCATTTGGGAGGCAGAGGCAGGA
GGATCTCTGAGTTTGAGGCCAGCCTGGTCTACAAAGCGAGTTCCAGGACAGCCAGGG
CTGTTCTTACACAAAGAAGAAACCTATCCTGAAAAACCAAATCCAACCAAACAAACAA
AAACTTTTTGCCATGGGAAAGCAGTGCTAGCTGTCCAGGGTACCAGGGGCTCTTTAGC
CCCCTGCCAAGCAGAATGGGCCATGCGTTCTAGGTGACCACCAGCTTGCATTTGTCCT
CCCACCTCAAGCTGCTCAGACCACAGGACTTAACACTTTCCTCATGGACACTATGGAG
AGTCCATTATGCTTAATTTTAATAAGTTTTTCTACTCTATAGATGCATACAATCTGCATT
TGCTGATCCACAGTGGGCATGGCAGCCCTTCCCAGTGCTGCTTTGAAGGGCTAGTAG
AATCGAGGGGAGGGATCTAGGGTTTTTGTTTTGTTTTGGGGATTTAATGCAGCAGGAG
CTAAGGAGACAACAGCCTTTCATTCTGCCAGAAAAATGGATGTGGTGTGGACACCCAT
AGGTGAGGGCTTAGGAAAATCATCCAAGCAACAGAATAAATTTTGAATTAGTTCTGTT
AGCCATGGAACAGAAGTTTAGGAAATAGGGTTCTAGATCACAGGCCCACTACTTGGT

GCTCACTCCTCTCATTCCATCTTCAGAGCCAGCAGCAGCAGGTGTTACACACACTCAA
AGGATAAAACATTGTTTGCCTCGTGCTCAGACTCTAGCGTTCTTGTAAGAATCAGAAGC
GAACCTATTCTGTTGGTAGTCCATGTACTGGCAAGTCTGTGTTGACACATTATTAAGTTG
TTTCCTTTTGAAATCTCAGAAGGCCTAGTTTATTTAGCATGTGTTTAATGTGAGGTGAGC
ACTGTGACAGAGGTGGATTTAGGAGGAAATGTGGTTATGTCTCTACCTGTGCTCTGGCA
TCTGTGGGGGAAAAAAAGGAATAACAGAGTTGAGGACATGGAATGTTTCAGGATATTTG
ATCACACTGTGCACTCTGAGCCTTTTACTGACAAACCCTGTTCCTGGTTGTGGTGTGGC
TCAGCCCTTAGCACACACCTTTAATCTCAAACAATGAAGATAAAGTTGGTTTGTAGGAGG
AAGCAGCCATGCTTGAAAGTGATGTCTAATTGAGTGGCAGACAAAGTGACCAATCAGAG
AAAGATGTGACAAATAGGATCTGCCTAACTTTCACAGGAAGAGGGGAAAGGGAAGCTAT
TTGAGGGGCAGTGCAGAGAGAGAGGGGGGGGGCATATTTACTGGGACAGTTGAACAG
AGACAGGTTGTAAAGAACAAGCTAGACACAGGTGAAGACAGAGCGAGCCAGAGAAGGA
GAAGGAGCCAGATTAGAACATACTGCCAAAGTTAGTCTGAGGCCAAGCAGAGAGCAATT
CAGGAGAGGCCCAGAGAGAAGCCAGACTGAATTCATCACCTTGGAGAGGTGTTTGAGC
CAGATCAGCTGAGTTGAACCAGCCAACCAGAGCTCAGAGAGAACTAGGAAGGGGTGAA
CTTATTCAGCAGCCAGCCTCGGCAGCTGAAAACATTCTAGGCTTTGATTAGATTGTACG
GAGGCTAGAAGCTTTCAGGACTGGGCCTAGGTTAGCAGACGGTGGAGGCAGTGAACCT
CAGAGGTGACAATTACTTCAAGAGAATAAAAGTTACATTTACAAGGGCATCCAGGGCTG
TGTATGTGCGTGTGCGCGTGCGCGCGCGCGTATGTGTGTGTGCGTGTGCGTGTGT
GTGTGTGTGTCAGTATGGCTGAACTGCTTTGAAGGATGAGTGTTTTCAGTGGGAAAGAG
TAGGCGAGCACGTGAAGGGCTTAGCCAAGGACAGGGAGAAGGACATCACTGAAGGCA
CCTGCAGTCTCGCTTATCAGCACAACACAGGCTGGAGCTGATAAACAGGGCTGGAGAG
GTGCAAGAACCAGAGCGCTGTGCCAGGGAGGCTGGGCTTTATCTGTAGCCTGGAGTCT
CGAGCTTTACGCTGCTAGGCTTTGCCCTTCTCCCTACCCACTTGGCCTTCAGGCTTGGG
ACTCTGCAGCCCACCTGCCTGTGCAGAGTGGTGCGAGTATCCAGTATCTGTTGCTTCAT
CCTTTCCATGCCCAGGACGAGCAGGTAGCTAATGCCCATTTGGACTCCTGACATAGTAC
ACATCGCATCCTAGGTGCAGGCTTTGTACCCCATACAGTGAGACATTCTCCAGCGAATG
CCAAGCAGGCACCCTTGGTTTTCTTTTAGCGTCTAAAGATACAGGTCTTTGTCTCACAAG
ACCACTCCACTTCAGATGCCTAACTAGAGTCTTTAGGTGTGATGCTCTTCTGTCTCCTTG
GTCCGATAAGTAAGGCCAGGGTCTGGGGATGCAGTTCTGTTTATCTCGCATGCATGGA
GCCCTGGGGTTGGTTCCCCAGCATCCCTGAAAACAGCTAGTCATGGAAACCTCTGATCT
TAGCGCTTAGGAAACACAGGCGAGGATCTGGATCCCAAGATCATCCTGGTCAACAGAG
AATTTGCGGCCAGCCTGGGCTACATGAGACCTCGTCTCTGGATGTGAGAGCCAGGGGC
CTGGGGAAATGGTTTAGTTGGTAAAGAGCCTTCTTCACAAACATGAGCACCTGGATTTG
GATTCCCAGGACCCACACTAAAAAATTGGGATGTGGTGGGATGTGTTTGTGACCCGAAC
TGGGCATGGGGGAGGTGTGGCGTAGAGCTTCCCGTGGAGAAAAGTGGGTTTCCAGAG
CTCATTGACCTGCCTGTGTAGCCAGCCAAGCAGTGAGCTCTGAGGTTAGGGAGAAACC
CTGTCTCAAAAATATGAGGTCGGAGAGGGATAGAGCCTCCATATACGCGTATACACATA
AGCACACACAGAGAACTTGTATGTACACACATGTACATGTATGCTTACACACAGGACTCA
GGAAAGTGCTTTACTTACATGTGACACTTAGTATGAGGATAGTATAAGAGTTCCAGCTAT
CTGGATGAAGAGGCGCACGTGACAAAGGACATGGGAAGGACAAGGAGCTTCCCAGCG
CTCCCCTGAACATCAGCCTCTAGACAGACACCTCCACATCTTCAGCAACCTGGAAGCTC
CCCAAGCCTATTGTGTGGGTATGACTAATATGGTTGACTAAATTGTCAGCCACTGGTTAA
ACATTCTACCTTAAGCCCTTCGCTAAGGTCAAGGGTAGGGCCTCCCATGATACAGTTGG
TTTCCCTGGCAGCTGGTCTGTATCCCGTGGCTACCCAGGTGTCTAAGAGTCAGGAAAAA
TATTGCTATCATTTCAGGAAATGGCAGAGGTCATAGGATCTCTGTGTCAGGAACTCCAG
CCAGGACCAGATACTGAAAATATTCTTACAGTGGCCCTATTACAAGGGAAATAACAA

Genomic and transcribed sequence of lncWDR59.

Genomic sequence of murine lncWDR59, located on the negative strand of chromosome 8 (mm9). In bolt, lncWDR59 RNA sequence (from RNA-seq and HotStart RACE-PCR). Underline: probe sequence; double underline: miR-103 predicted binding site.

Supplementary References

1. Hartmann P*, et al.* Endothelial Dicer promotes atherosclerosis and vascular inflammation by miRNA-103-mediated suppression of KLF4. *Nat Commun* **7**, 10521 (2016).

2. Guttman M*, et al.* Chromatin signature reveals over a thousand highly conserved large non-coding RNAs in mammals. *Nature* **458**, 223-227 (2009).

3. Bartel DP. MicroRNAs: Target Recognition and Regulatory Functions. *Cell* **136**, 215-233 (2009).

4. Cambronne XA, Shen R, Auer PL, Goodman RH. Capturing microRNA targets using an RNA-induced silencing complex (RISC)-trap approach. *Proc Natl Acad Sci U S A* **109**, 20473-20478 (2012).

5. Zhou Z*, et al.* Lipoprotein-derived lysophosphatidic acid promotes atherosclerosis by releasing CXCL1 from the endothelium. *Cell Metab* **13**, 592-600 (2011).

6. Bellucci M, Agostini F, Masin M, Tartaglia GG. Predicting protein associations with long noncoding RNAs. *Nat Methods* **8**, 444-445 (2011).
